# A Lightweight PCB Defect Detection Method Based on Heterogeneous Feature Enhancement and Discrepancy-Guided Fusion

**DOI:** 10.3390/s26185984

**Published:** 2026-09-21

**Authors:** Yujie Pei, Xuehong Gao, Shenyuan Gao, Yongchang Zhang, Ying Liu, Guozhong Huang, Lili Lei

**Affiliations:** 1 Research Institute of Macro-Safety Science, University of Science and Technology Beijing, Beijing 100083, Chinahjxhuanggz@ustb.edu.cn (X.G.);; 2 Key Laboratory of Automotive Chip Testing and Evaluation, State Administration for Market Regulation, Beijing National New Energy Vehicle Technology Innovation Center Co., Ltd., Beijing 100176, China; 3 Department of Computer Science and Technology, Tsinghua University, Beijing 100084, China

**Keywords:** heterogeneous feature enhancement, industrial defect detection, lightweight detection, printed circuit board

## Abstract

Accurate detection of small and weak defects is essential for ensuring the manufacturing quality and operational reliability of printed circuit boards (PCBs). Existing detectors, however, remain constrained by insufficient fine-grained feature representation, interference from repetitive conductive backgrounds, localization instability, and excessive computational complexity. To address these limitations, a lightweight defect detection model, termed Compact Recalibration and Fusion YOLO (CRF-YOLO), is developed based on YOLO11n. C3k2-Lite integrates partial-channel spatial modeling with cross-stage feature aggregation, reducing redundant computation while retaining essential defect information. The Residual Feature Fusion Attention module (RFFA) performs heterogeneous defect-evidence decomposition by jointly encoding positional, boundary, connectivity, and texture cues. Independently gated evidence aggregation and dual-dimensional feature recalibration strengthen weak contour interruptions, abnormal conductive connections, and subtle texture disturbances embedded in complex circuit backgrounds. The Residual Cross-Fusion module (RCF) establishes discrepancy-guided dual-stream feature reconciliation between the original and attention-enhanced representations. Location-adaptive feature selection and structure-aware detail reconstruction preserve low-amplitude defect cues while selectively incorporating discriminative information. Shape-NWD is adopted to improve the localization stability of small, elongated, and geometrically irregular defects. Experimental results show that CRF-YOLO achieves a Precision of 95.53%, a Recall of 91.26%, an mAP@0.5 of 94.46%, and an mAP@0.5:0.95 of 51.74%, with 2.175 M parameters and 6.0 GFLOPs. Compared with representative mainstream object detectors, the proposed model delivers superior overall detection performance while maintaining more favorable lightweight characteristics. A browser-based inspection interface is also implemented to support image uploading, automated defect detection, and result visualization, demonstrating the practical deployment potential of the proposed approach.

## 1. Introduction

The rapid development of information technology has established printed circuit boards (PCBs) as fundamental components of modern electronic systems. PCBs provide electrical connections and mechanical support for electronic components and are extensively used in consumer electronics, communication equipment, industrial control systems, medical devices, and automotive electronics. Their manufacturing quality directly affects the reliability and operational stability of electronic products. Therefore, accurate PCB defect detection is of considerable importance for electronic manufacturing and quality control [1].

The increasing miniaturization and integration of electronic devices have resulted in more compact PCB layouts, narrower conductive traces, and smaller component spacing. These changes increase the risk of manufacturing defects and make them more difficult to identify. Common PCB defects include missing holes, mouse bites, open circuits, short circuits, spurs, and spurious copper [2,3]. Although these defects usually occupy only a small area of the PCB image, they may interrupt electrical connections, generate unintended conductive paths, or affect component installation. Undetected defects may subsequently lead to unstable circuit operation, electronic product failure, and potential safety risks.

Traditional PCB inspection mainly relies on manual visual examination and automated optical inspection based on conventional image-processing methods. Manual inspection is inefficient and susceptible to operator experience and fatigue, while traditional automated methods are often sensitive to illumination variation, image misalignment, background interference, and changes in defect appearance [1,3]. With the development of deep learning, object detection algorithms have gradually become important tools for PCB inspection. In particular, the You Only Look Once (YOLO) series has attracted considerable attention because of its end-to-end architecture and favorable balance between detection accuracy and computational efficiency [4,5]. However, existing methods still face several practical limitations. PCB defects are generally small and have weak visual features, which may be lost during repeated downsampling. Similar defect categories are also difficult to distinguish in densely distributed circuit backgrounds. Moreover, improvements based on complex attention mechanisms and multi-scale structures often introduce additional parameters and computational costs, making it difficult to simultaneously achieve high detection accuracy and lightweight deployment [5,6]. Conventional bounding-box regression methods are also sensitive to slight positional deviations in small objects, which may reduce localization stability [7].

To address these challenges, this study proposes Compact Recalibration and Fusion YOLO (CRF-YOLO), a lightweight PCB defect detector built upon YOLO11n. First, a lightweight feature extraction module, termed C3k2-Lite, is developed by combining partial-channel spatial modeling with cross-stage feature aggregation, thereby reducing redundant computation while preserving fine-grained defect cues. Second, a Residual Feature Fusion Attention (RFFA) module is designed following the principle of heterogeneous defect-evidence decomposition. Specifically, RFFA explicitly models spatial coordinates, boundary gradients, anisotropic connectivity patterns, and local texture statistics, and subsequently performs independently gated evidence aggregation and dual-dimensional feature recalibration. This mechanism enables the network to selectively amplify weak contour breaks, abnormal trace connectivity, and subtle texture disturbances embedded in repetitive PCB backgrounds. Third, a Residual Cross-Fusion (RCF) module is introduced as a discrepancy-guided dual-stream feature reconciliation mechanism. By jointly modeling the original P3 feature, its attention-enhanced counterpart, and their response discrepancy, RCF performs location-adaptive feature selection and structure-aware detail reconstruction. Consequently, low-amplitude yet informative defect signals are preserved while discriminative enhancements are selectively injected, alleviating the over-suppression of tiny defects caused by aggressive attention recalibration. In addition, the existing Shape-NWD loss is adopted as the bounding-box regression objective to improve localization accuracy for small and geometrically diverse defects. Through the coordinated optimization of lightweight feature extraction, heterogeneous evidence modeling, discrepancy-aware feature reconciliation, and shape-sensitive regression, CRF-YOLO improves small-defect detection while maintaining low model complexity.

The architectural contribution of CRF-YOLO lies in the resolution-aware allocation of C3k2-Lite and the coupled enhancement–reconciliation pathway formed by RFFA and RCF. Rather than treating lightweight transformation, attention recalibration, and feature fusion as independent plug-in operations, the proposed architecture links them according to the successive requirements of computational reduction, defect-evidence enhancement, and weak-information recovery. Shape-NWD is employed as an existing complementary regression objective and is not claimed as a newly developed loss function.

The remainder of this paper is organized as follows. Section 2 reviews the current state of research on vision-based PCB defect detection, lightweight detection networks, attention mechanisms, feature fusion, and bounding-box regression. Section 3 presents the overall architecture of CRF-YOLO and describes the proposed C3k2-Lite, RFFA, RCF. Experimental settings, ablation studies, comparative experiments, and result discussions are presented in Section 4. Finally, Section 5 concludes this study and outlines possible directions for future research.

## 2. Related Work

Recent advances in deep learning have substantially improved the accuracy and efficiency of vision-based PCB defect inspection. Current studies mainly focus on optimizing one-stage detectors through lightweight convolution, attention-based feature enhancement, multi-scale feature fusion, and improved bounding-box regression. These approaches have reduced the dependence on manually designed inspection rules and improved the recognition of small defects in complex circuit backgrounds. However, PCB defects often contain limited visual information and exhibit substantial intra-class variation and inter-class similarity. Meanwhile, practical inspection systems require low computational complexity and stable localization performance. Existing methods therefore still face difficulties in simultaneously preserving fine-grained features, suppressing background interference, reducing model complexity, and accurately locating small defect regions.

### 2.1. PCB Defect Detection Models

In recent years, the YOLO family has become one of the principal frameworks for PCB defect detection because of its end-to-end inference process and real-time capability. Most related studies have improved existing YOLO architectures according to the small size, weak appearance, and complex background characteristics of PCB defects.

Xiao et al. [8] proposed CDI-YOLO based on YOLOv7-tiny. Coordinate attention was introduced into the backbone and neck to enhance spatial feature representation, while depthwise separable convolution was employed to reduce computational cost. Inner-CIoU was further adopted to accelerate bounding-box regression. The resulting model achieved a favorable balance among detection accuracy, speed, and model size. Gao et al. [9] developed YOLOv5_ES for small PCB surface defects. The method removed detection branches designed for medium and large objects and retained a small-object detection head. It also incorporated SPD-Conv to reduce spatial-information loss and an efficient multi-scale attention mechanism to aggregate contextual features.

More recent studies have increasingly combined lightweight structures with PCB-specific feature enhancement. Wei et al. [10] proposed PCB-YOLO based on YOLOv8n. Their method introduced a compact feature extraction module combining SCConv and C2f, together with an attention-based multi-scale fusion structure. An auxiliary-boundary IoU loss was also used to improve the regression of small defects. Yu et al. [11] developed a lightweight PCB detector by integrating GhostNet, depthwise separable convolution, a Swin-Transformer-based feature block, and a bidirectional feature pyramid. These components were used to reduce redundant computation while maintaining global-context modeling and multi-scale representation. Such studies demonstrate that recent PCB detection research has gradually shifted from direct model transplantation toward coordinated optimization of the backbone, neck, attention mechanism, and localization objective.

### 2.2. Lightweight and Attention-Based Detection Methods

Lightweight network design is essential for deploying PCB defect detectors on embedded inspection equipment and edge-computing platforms. Common strategies include replacing standard convolution with depthwise or grouped convolution, adopting compact backbone networks, reducing redundant feature channels, and redesigning repeated feature extraction blocks.

Li et al. [12] proposed SCF-YOLO, which used MobileNet as the backbone and introduced learnable weighted fusion in the neck. A synthesized C2f module was developed to strengthen high-level semantic representation while maintaining low computational complexity. Li et al. [13] proposed EL-PCBNet for efficient PCB inspection. The model contained a C3-Faster module for reducing redundant computation, a C3F-SimAM module for enhancing small-defect features, and an improved spatial pyramid structure for associating information at different scales. Li et al. [14] subsequently developed GS-YOLO, in which a C3Ghost-based module was used to reduce model complexity and a global-attention spatial pyramid module was employed to combine local saliency with contextual information. These studies indicate that lightweight design is no longer limited to simply replacing standard convolution; recent methods increasingly attempt to maintain feature quality through structural reparameterization, attention enhancement, and selective multi-scale processing.

Attention mechanisms provide another important approach for improving defect representation. The squeeze-and-excitation block proposed by Hu et al. [15] recalibrates feature channels according to global channel statistics. Woo et al. [16] extended this concept through the Convolutional Block Attention Module, which sequentially applies channel and spatial attention. Wang et al. [17] proposed Efficient Channel Attention, using local cross-channel interaction without dimensionality reduction to limit parameter overhead. Hou et al. [18] introduced Coordinate Attention, which decomposes spatial aggregation into two directional operations and embeds positional information into channel attention. These classical mechanisms have been widely incorporated into industrial defect detectors because they can be inserted into convolutional networks with limited structural modification. Nevertheless, their effectiveness depends strongly on the insertion position, feature-map resolution, and visual properties of the target. Generic attention mechanisms may emphasize dominant responses, whereas weak boundary interruptions and subtle connectivity changes require more task-oriented feature modeling.

### 2.3. Multi-Scale Feature Fusion and Bounding-Box Regression

Multi-scale feature fusion is particularly important for PCB inspection because defects of different categories may occupy substantially different areas and exhibit different levels of visual saliency. Shallow features retain detailed spatial information, whereas deep features provide stronger semantic discrimination. Effective detection therefore requires the controlled interaction of features from multiple network stages.

The feature pyramid network and its subsequent variants established the basic framework for combining high-level semantic information with low-level spatial details. Tan et al. [19] proposed the weighted bidirectional feature pyramid network in EfficientDet, allowing features from different scales to be fused repeatedly with learnable weights. Similar bidirectional and weighted fusion strategies have since been adopted in PCB detection models. For example, the learnable fusion mechanism in SCF-YOLO [12], the bidirectional feature pyramid used by Yu et al. [11], and the multi-scale attention fusion adopted in PCB-YOLO [10] all aim to improve the transmission of weak defect information.

However, repeated resampling, concatenation, and convolution may also introduce redundant features or attenuate useful shallow responses. Consequently, recent studies increasingly emphasize selective fusion, residual transmission, and adaptive weighting rather than direct feature accumulation.

Bounding-box regression directly affects the localization accuracy of small PCB defects. Conventional IoU-based losses evaluate overlap between predicted and ground-truth boxes, but their regression behavior may become unstable when the target occupies only a few pixels. Ma and Xu [20] proposed MPDIoU, which measures the distances between corresponding corner points and incorporates overlap, center displacement, and width–height differences into a relatively simple formulation. Luo et al. [21] proposed Unified-IoU, which dynamically shifts the optimization focus from low-quality to high-quality predicted boxes during training. For tiny-object detection, NWD models bounding boxes as Gaussian distributions and measures their similarity using normalized Wasserstein distance, thereby reducing sensitivity to small coordinate deviations [7]. Zhang and Zhang [22] further investigated the influence of the intrinsic shape and scale of bounding boxes on regression. They proposed Shape-IoU and, within the same study, formulated Shape-NWD for tiny-object detection by incorporating shape-related weighting into the normalized Wasserstein representation. Shape-NWD is therefore an existing localization method rather than a loss developed in the present study. Its suitability for PCB defects must be determined through task-specific comparative experiments, because the effectiveness of a regression loss depends on the object-size distribution, defect geometry, detector architecture, and training strategy.

Overall, previous studies have demonstrated the effectiveness of lightweight convolution, attention mechanisms, multi-scale fusion, and improved regression losses for PCB defect detection. Nevertheless, existing lightweight blocks mainly focus on reducing convolutional redundancy, while generic attention mechanisms generally estimate saliency from channel statistics or broad spatial responses without explicitly representing the position, boundary, connectivity, and texture characteristics of PCB defects. Conventional feature-fusion methods commonly employ direct addition, concatenation, or scalar weighting, but seldom examine the response discrepancy between an original feature and its attention-enhanced counterpart. Consequently, a gap remains in linking efficient feature transformation, defect-oriented enhancement, and weak-information recovery within a unified lightweight detector. CRF-YOLO addresses this gap through the resolution-aware deployment of C3k2-Lite and the functionally coupled RFFA–RCF pathway.

## 3. Methodology

**Figure 1:**
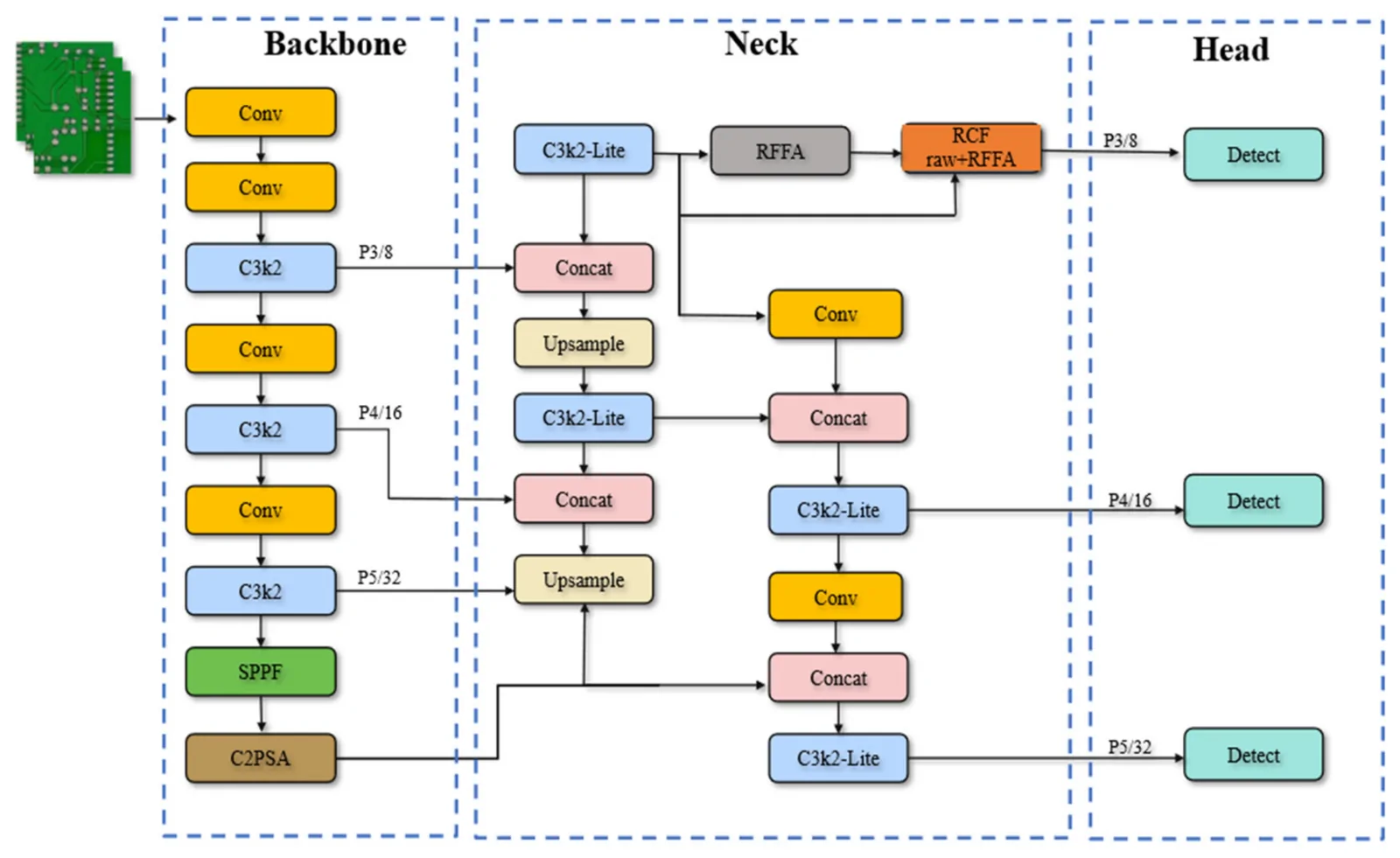
CRF-YOLO model architecture. Conv denotes convolution; SPPF denotes Spatial Pyramid Pooling–Fast; C2PSA denotes a C2 block with position-sensitive attention; RFFA denotes Residual Feature Fusion Attention; and RCF denotes Residual Cross-Fusion. P3/8, P4/16, and P5/32 represent feature levels with downsampling strides of 8, 16, and 32, respectively. Different colors distinguish module types, solid arrows indicate feature-flow directions, and dashed boxes delimit the backbone, neck, and detection head.

CRF-YOLO is developed from YOLO11n to address the weak representation and unstable localization of small PCB defects under lightweight constraints. The original backbone and three-scale detection framework are retained to preserve computational efficiency. On this basis, the neck and high-resolution detection branch are redesigned to enhance fine-grained defect perception without introducing excessive computational overhead. The overall architecture of CRF-YOLO is illustrated in Figure 1.

C3k2-Lite is selectively introduced into the neck as an efficient partial-channel feature transformation mechanism. By combining partial spatial computation with cross-stage aggregation, it suppresses redundant operations while retaining essential semantic information. For the high-resolution P3 branch, RFFA performs heterogeneous defect-evidence modeling by jointly encoding positional, boundary, connectivity, and texture cues. Through independently gated aggregation and spatial–channel recalibration, RFFA strengthens subtle structural anomalies embedded in repetitive conductive backgrounds.

RCF is subsequently employed as a discrepancy-guided dual-stream feature reconciliation mechanism. It adaptively coordinates the raw P3 representation and its RFFA-enhanced counterpart, preserving low-amplitude defect cues while selectively injecting discriminative information. An asymmetric routing strategy is further adopted: the enhanced P3 feature is delivered to the small-object detection head, whereas the original P3 feature continues to construct the P4 and P5 branches, thereby maintaining stable multi-scale feature propagation. During training, Shape-NWD provides shape-sensitive distributional regression, improving the localization robustness of small, elongated, and irregular defects. These components jointly enable CRF-YOLO to achieve enhanced defect discrimination and localization accuracy with low model complexity.

The modules are organized as a functionally coupled processing chain rather than as parallel architectural additions. C3k2-Lite is deployed in the medium- and high-level fusion paths to reduce redundant computation, while the original C3k2 structure is retained on the high-resolution P3 branch to protect fine spatial information. RFFA subsequently converts the P3 feature into a defect-oriented representation by jointly modeling position, boundary, connectivity, and texture evidence. Because attention enhancement may attenuate weak but informative responses, RCF does not directly replace the original feature; instead, it compares the raw and enhanced representations, uses their discrepancy to control feature selection, and reconstructs potentially weakened structural details. The reconciled representation is delivered only to the P3 detection head, whereas the raw feature continues to construct the P4 and P5 branches. This constrained pathway establishes the sequence of efficient representation, defect-oriented enhancement, and discrepancy-guided information recovery, while Shape-NWD complements the architecture at the regression level during training.

### 3.1. C3k2-Lite Module

**Figure 2:**
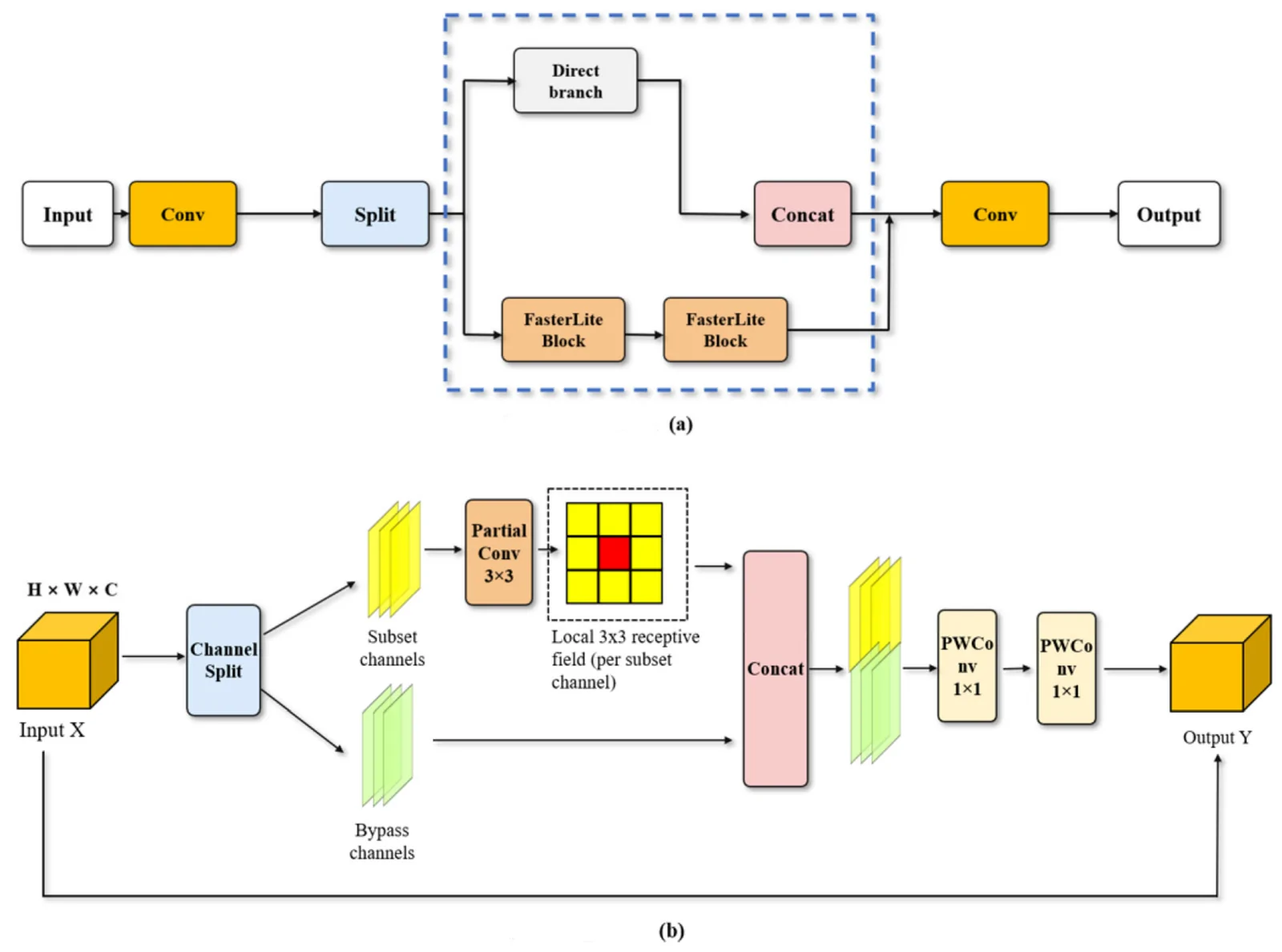
Structures of the C3k2-Lite module (**a**) and the FasterLiteBlock (**b**). H, W, and C denote the height, width, and number of channels of the feature map, respectively; PWConv denotes pointwise convolution. The arrows indicate feature-flow directions, and the dashed box identifies the C3k2-Lite module.

The C3k2 module in YOLO11n employs cross-stage feature aggregation to promote feature reuse and gradient propagation [23]. However, repeatedly applying spatial convolution to all hidden channels introduces unnecessary computation in the neck. Recent lightweight architectures have further shown that efficient network design should consider operator complexity, memory access, and practical inference latency rather than relying exclusively on parameter count or FLOPs [24,25]. Related work on asymmetric convolution blocks has shown that strengthening the kernel skeleton can improve feature representation [26]. Accordingly, C3k2-Lite is developed as an asymmetric partial-channel transformation module. It preserves the split–transform–aggregate topology of C3k2, while replacing its internal bottleneck with the proposed FasterLiteBlock, as illustrated in Figure 2. The functional composition of FasterLiteBlock is summarized in Table 1.

Given an input feature map X, a 1×1 convolution first aligns the channel dimension, and the resulting feature is divided into a direct branch $X_{a\;}$ and a transformation branch $X_{b}$:

(1) $$\left[X_{1},X_{2}\right]=Split({Conv}_{1\times 1}(X))$$

The direct branch provides an information-preserving pathway, whereas $X_{b}$ is progressively processed by FasterLiteBlocks. Within each block, the input feature F is partitioned into a spatially processed group $F_{p}$ and a bypass group $F_{b}$. Following the Partial Convolution principle [27], only $F_{p}$ undergoes a 3×3 spatial convolution:

(2) $$F_{S}=\left[{Conv}_{3\times 3}(F_{p}),F_{b}\right]$$

Two pointwise convolutions subsequently restore cross-channel interaction. With a learnable layer-scaling coefficient γ, the block output is expressed as:

(3) $$F_{i}=F_{i-1}+\gamma_{i}{Conv}_{1\times 1}^{\left(1\right)}[\delta ({Conv}_{1\times 1}^{\left(1\right)}(F_{s}^{i-1}))]$$

where $F_{i-1}$ and $F_{i}$ denote the input and output of the i-th FasterLiteBlock, respectively, δ(⋅) represents the SiLU activation function, and $\gamma_{i}$ is a learnable layer-scaling coefficient. When the shortcut connection is disabled, the identity term $F_{i-1}$ is omitted. The pointwise convolutions compensate for the limited channel interaction of Partial Convolution. Following the residual-learning principle [28], the shortcut path enables FasterLiteBlock to learn a feature refinement relative to its input rather than reconstructing the complete representation. This formulation facilitates gradient propagation and prevents the lightweight transformation from excessively modifying the original feature distribution.

After sequential transformation, the direct feature, initial transformation feature, and intermediate block outputs are aggregated:

(4) $$Y={Conv}_{1\times 1}[Concat(X_{1},X_{2},F_{1},\ldots ,F_{n})]$$

In CRF-YOLO, C3k2-Lite is applied to the medium- and high-level feature-fusion paths, whereas the original C3k2 is retained in the high-resolution P3 branch. This resolution-aware computational allocation concentrates lightweight processing on semantically enriched features while preserving detailed spatial information for small-defect detection. The hidden-channel expansion ratio and partial-convolution ratio are set to 0.1875 and 0.125, respectively. Consequently, C3k2-Lite reduces redundant spatial computation through partial-channel modeling, information-preserving transmission, and cross-stage aggregation.

Unlike C3Ghost, which generates additional features through inexpensive linear operations, and C2fCIB, which relies on compact inverted blocks, C3k2-Lite reduces redundancy by restricting spatial convolution to a selected channel subset while retaining an explicit information-preserving bypass. Its novelty also lies in its resolution-aware deployment: lightweight transformation is concentrated in the medium- and high-level fusion paths, whereas the original high-resolution P3 extraction structure is retained to avoid weakening small-defect details.

### 3.2. Residual Feature Fusion Attention Module

**Figure 3:**
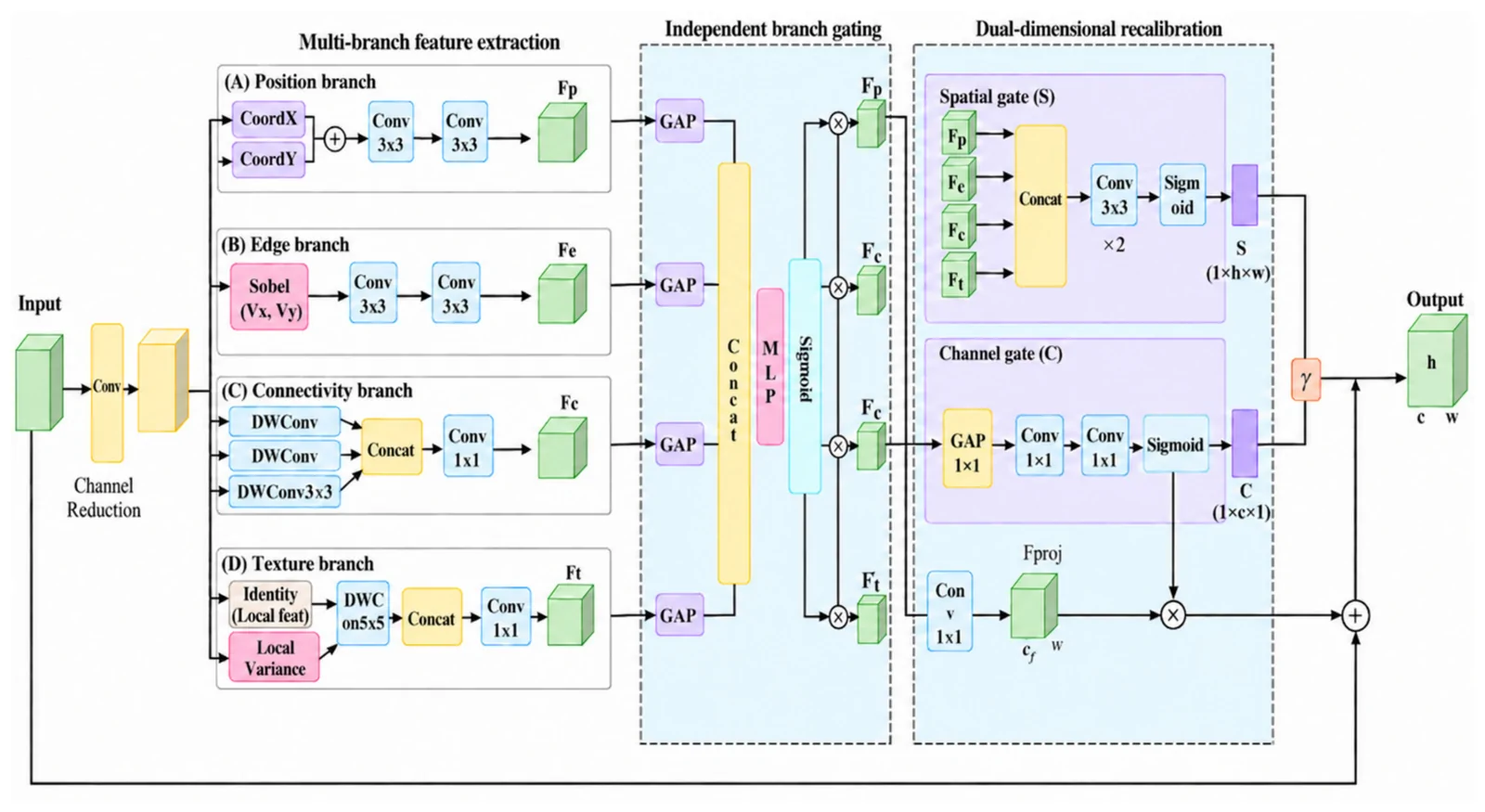
Structure of the Residual Feature Fusion Attention module. CoordX and CoordY denote normalized horizontal and vertical coordinate maps, respectively; ∇x and ∇y denote the horizontal and vertical Sobel gradients. Conv, DWConv, GAP, and MLP denote convolution, depthwise convolution, global average pooling, and multilayer perceptron, respectively. S and C denote the spatial and channel attention maps, respectively; γ is a learnable scaling coefficient; and h, w, and c denote feature-map height, width, and channel count. The circled C denotes concatenation, whereas ⊗ and ⊕ denote element-wise multiplication and addition, respectively. Arrows indicate feature-flow directions, dashed boxes delimit processing stages, and colors distinguish different functional components rather than quantitative values.

Small PCB defects are commonly embedded in repetitive conductive traces and exhibit heterogeneous visual manifestations, including positional deviations, contour interruptions, abnormal connectivity, and local texture disturbances. Generic channel or spatial attention may overemphasize dominant responses while overlooking these low-amplitude structural anomalies. Therefore, the Residual Feature Fusion Attention (RFFA) module is introduced into the high-resolution P3 branch as a heterogeneous defect-evidence modeling mechanism. As illustrated in Figure 3, RFFA performs multi-cue decomposition, non-exclusive evidence gating, dual-dimensional recalibration, and identity-preserving enhancement.

Given an input feature map X, a 1 × 1 convolution first generates a compact representation $F_{0}$. The reduced feature is subsequently processed by four parallel evidence branches:

(5) $$F_{0}=\emptyset_{r}(X)$$

(6) $$F_{p}=\emptyset_{p}(\left|F_{0},C_{x},C_{y}\right|)$$

(7) $$F_{e}=\emptyset_{c}(F_{0}+\alpha S(F_{0}))$$

(8) $$F_{c}=\emptyset_{c}([D_{h}\left(F_{0}\right),D_{v}{\left(F_{0}\right),D}_{d}(F_{0})])$$

(9) $$F_{t}=\emptyset_{t}([F_{0},D_{5}\left(F_{0}\right),V(F_{0})])$$

where $C_{x\;}$ and $C_{y}$ denote normalized coordinate maps; $S\left(\cdot \right)$ represents the Sobel gradient response; α is a bounded learnable coefficient; $D_{h}$, $D_{v}$, and $D_{d}$ denote horizontal, vertical, and dilated depthwise convolutions, respectively; and $V\left(\cdot \right)$ represents local variance. Explicit coordinate encoding preserves location-dependent information [29], while dilated convolution enlarges the effective receptive field without reducing spatial resolution [30]. The four heterogeneous evidence branches of RFFA are summarized in Table 2.

Unlike softmax-based competitive selection, RFFA adopts non-exclusive evidence gating, allowing several complementary cues to remain active simultaneously. Adaptive multi-branch selection has been shown to improve the coordination of heterogeneous receptive fields [31], while cross-dimensional attention can strengthen interactions between spatial and channel representations [32]. Based on these principles, RFFA generates four independent branch gates:

(10) $$d=[GAP\left(F_{p}\right),AP\left(F_{e}\right),AP\left(F_{c}\right),AP\left(F_{t}\right)]$$

(11) $$g_{i}=\beta +(1-\beta )\sigma ({MLP(d)}_{i})$$

(12) $$F_{f}=\frac{1}{4}\sum_{i\in \{p,e,c,t\}}g_{i}F_{i}$$

where $g_{i}$ denotes the gate of the i-th evidence branch and β is a nonzero lower bound. This formulation prevents weak but informative evidence from being completely suppressed and enables joint activation of correlated cues, such as boundary discontinuity and abnormal connectivity.

The fused representation is subsequently recalibrated in the spatial and channel dimensions. The spatial gate is estimated from the concatenated evidence features, whereas the channel gate is generated from the globally pooled fused representation. Channel and spatial attention provide complementary feature-selection capabilities [16]. The final output is formulated as:

(13) $$A_{s}=\eta +(1-\eta )(\phi_{s}[F_{p},F_{e},F_{c},F_{t}])$$

(14) $$Y=X+\gamma \left[\phi_{s}(F_{f})⨀A_{c}⨀A_{s}\right]$$

where $A_{s}$ and $A_{c\;}$ denote the spatial and channel attention maps, respectively; η is the lower bound of the spatial gate; γ is a learnable scaling coefficient; and ⨀ denotes element-wise multiplication. The scaled shortcut follows the residual-learning principle [28] and keeps the module close to identity mapping during early optimization.

RFFA is deployed only on the P3 detection branch, concentrating defect-oriented evidence recalibration on the feature map with the highest spatial resolution. Its output is subsequently reconciled with the raw P3 representation through RCF. Consequently, RFFA enhances weak structural anomalies while avoiding extensive perturbation of the original multi-scale feature pyramid.

Unlike SE and ECA, which primarily model channel relationships, or CBAM and Coordinate Attention, which derive general spatial–channel saliency, RFFA explicitly decomposes PCB defect evidence into position, boundary, connectivity, and texture responses. Its independently generated non-exclusive gates allow multiple correlated cues to remain active simultaneously rather than forcing them to compete for a single dominant response. Therefore, the contribution of RFFA lies in task-oriented evidence decomposition and coordinated multi-cue recalibration rather than the direct reuse of a generic attention mechanism.

### 3.3. Residual Cross-Fusion Module

**Figure 4:**
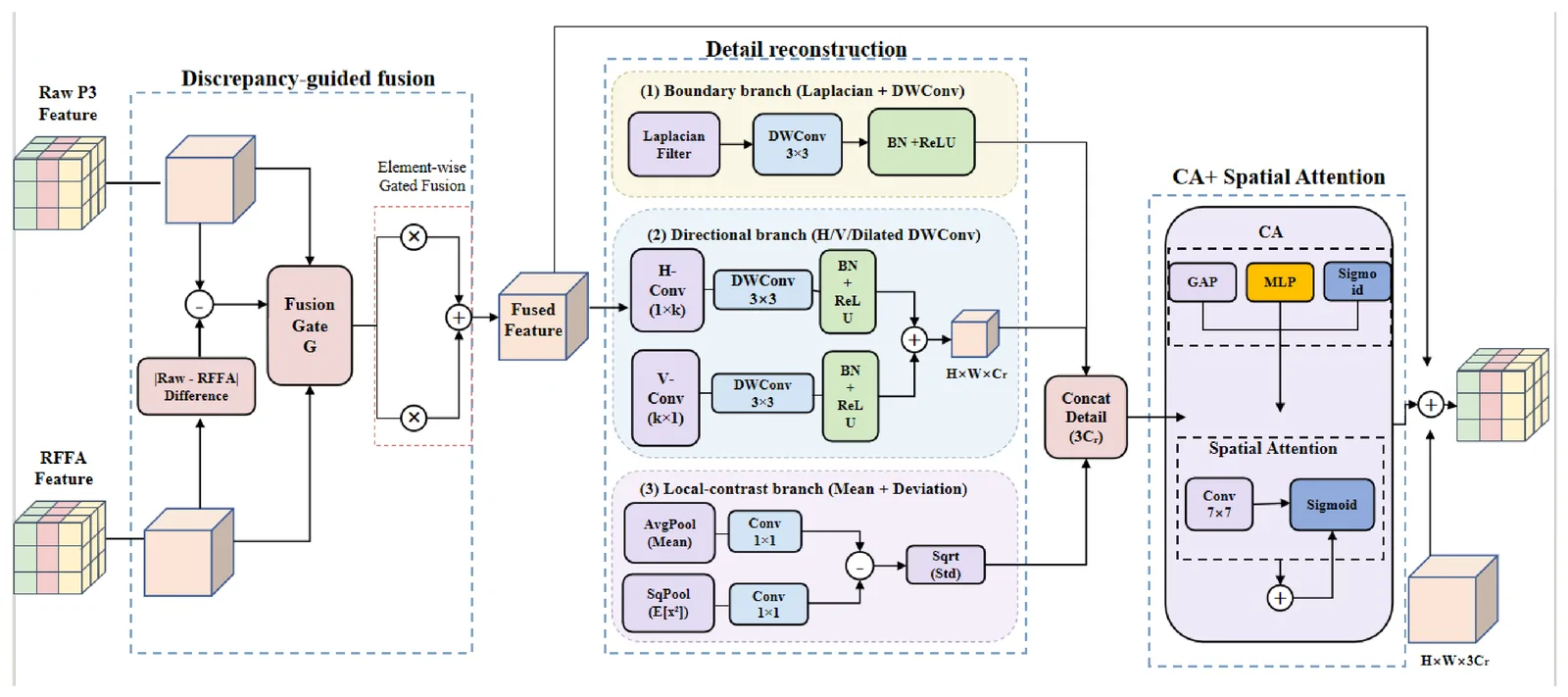
Structure of the Residual Cross-Fusion module. P3 denotes the feature level with a downsampling stride of 8, and RFFA denotes Residual Feature Fusion Attention. DWConv, BN, ReLU, AvgPool, SqPool, Std, Sqrt, CA, GAP, and MLP denote depthwise convolution, batch normalization, rectified linear unit, average pooling, mean-square pooling, standard deviation, square root, Coordinate Attention, global average pooling, and multilayer perceptron, respectively. H-Conv and V-Conv denote horizontal and vertical convolutions, respectively; H, W, and Cr denote feature-map height, width, and reduced channel count. The circled −, ×, and + symbols denote element-wise subtraction, multiplication, and addition, respectively. Arrows indicate feature-flow directions, dashed boxes delimit processing stages, and colors distinguish functional components rather than quantitative values.

Although RFFA improves the discriminability of the P3 feature, attention recalibration may attenuate low-response cues whose appearance resembles normal conductive structures. Directly replacing the raw P3 representation with the enhanced feature may therefore suppress tiny contour breaks or weak connectivity anomalies. To address this issue, RCF is developed as a discrepancy-guided dual-stream feature reconciliation mechanism. As shown in Figure 4, it combines discrepancy-conditioned spatial arbitration with structure-aware detail reconstruction, enabling discriminative enhancement without sacrificing the original fine-grained information.

Let $X_{r}$ and $X_{e}$ denote the raw P3 feature and the RFFA-enhanced feature, respectively. RCF explicitly calculates their response discrepancy and generates a spatial arbitration map:

(15) $$∆X=\left|X_{r}-X_{e}\right|$$

(16) $$G=\sigma (\emptyset_{g}(\left[X_{r},X_{e},∆X\right]))$$

(17) $$F_{m}=G⨀X_{e}+(1-G)⨀X_{r}$$

where $\phi_{g}\left(\cdot \right)$ denotes the gate-generation transformation and G determines the contribution of each stream at every spatial position. Spatially adaptive fusion can suppress conflicting responses by assigning location-dependent weights rather than applying uniform feature accumulation [33]. Spatial-aware modulation has also been shown to improve the selection of informative regions in object detection [34].

The reconciled feature $F_{m}$ is projected into a compact representation $F_{0}$, from which three complementary detail branches are constructed:

(18) $$F_{b}=\emptyset_{b}(F_{0}+\alpha L(F_{0}))$$

(19) $$F_{d}=\emptyset_{d}([D_{h}\left(F_{0}\right),D_{v}{\left(F_{0}\right),D}_{a}(F_{0})])$$

(20) $$F_{l}=\emptyset_{l}([F_{0},\left|F_{0}-\mu \left(F_{0}\right)\right|,{\left(F_{0}-\mu \left(F_{0}\right)\right)}^{2}])$$

where $L\left(\cdot \right)$ denotes the Laplacian response; $D_{h}$, $D_{v}$, and $D_{a}$ represent horizontal, vertical, and dilated depthwise convolutions; and $\mu \left(\cdot \right)$ is the local mean.

The structure-aware detail branches of RCF are summarized in Table 3. The three responses are concatenated to form a unified detail representation. Coordinate Attention [18] then introduces directional and positional recalibration, while a spatial gate suppresses background responses. The final output is formulated as:

(21) $$F_{s}=\emptyset_{f}\left(\left[F_{b},F_{d},F_{l}\right]\right)$$

(22) $$Y=\emptyset_{s}\left(F_{m}\right)+\gamma \phi_{o}(A_{ca}(F_{s})⨀A_{s})$$

where $\phi_{s}\left(\cdot \right)$ is the shortcut projection, $A_{ca}\left(\cdot \right)$ denotes Coordinate Attention, $A_{s}$ is the spatial weighting map, and γ is a learnable scaling coefficient. This conservative detail-injection strategy performs controlled correction rather than complete feature replacement, allowing RCF to preserve low-amplitude defect evidence while recovering boundary, continuity, and local-contrast responses.

The RCF output is delivered only to the P3 detection head, whereas P4 and P5 remain constructed from the raw P3 feature. This asymmetric enhancement routing confines specialized reconstruction to the small-object branch and maintains stable multi-scale feature propagation.

RCF differs from conventional addition, concatenation, or scalar-weighted pyramid fusion in two respects. It reconciles two representational states of the same P3 feature—the original feature and its RFFA-enhanced counterpart—rather than directly merging features from different resolutions. Moreover, their absolute response discrepancy explicitly participates in spatial arbitration, after which boundary, directional-continuity, and local-contrast cues are selectively reconstructed. This discrepancy-conditioned reconciliation and asymmetric routing constitute the principal architectural distinction of RCF.

### 3.4. Shape-NWD for Bounding-Box Regression

PCB defects are typically small and geometrically diverse. Elongated defects, such as open circuits and short circuits, are particularly sensitive to minor coordinate deviations. For these targets, IoU-based losses may vary sharply even when the predicted box undergoes only a slight displacement. Although CIoU jointly considers overlap, center distance, and aspect-ratio consistency [35], its optimization remains dependent on the overlap between bounding boxes. In contrast, NWD models bounding boxes as two-dimensional Gaussian distributions and provides a continuous similarity measure under weak-overlap and non-overlap conditions [7]. Shape-NWD further introduces shape-aware directional weighting [22], forming a geometry-adaptive distributional regression mechanism suitable for small and anisotropic PCB defects.

Let the predicted bounding box and ground-truth bounding box be denoted by $B_{p}=(x_{p},y_{p},w_{p},h_{p})$ and $B_{g}=(x_{g},y_{g},w_{g},h_{g})$, respectively, where $\left(x,y\right)$ denotes the center coordinates and w and h denote the width and height. Following the shape-weighting strategy of Shape-IoU, the horizontal and vertical weights are calculated as:

(23) $$W_{w}=\frac{2w_{g}^{s}}{w_{g}^{s}+h_{g}^{s}},W_{h}=\frac{2h_{g}^{s}}{w_{g}^{s}+h_{g}^{s}}$$

where the scale factor controls the sensitivity of the directional weights to the aspect ratio. These weights assign greater regression emphasis to the dominant geometric direction of the target.

The shape-aware center-distance term is calculated as:

(24) $$D_{shape}=W_{w}{\left(x_{p}-x_{g}\right)}^{2}+W_{h}{\left(y_{p}-y_{g}\right)}^{2}$$

The width and height discrepancies are expressed as:

(25) $$S_{shape}=\frac{W_{w}}{4}{\left(w_{p}-w_{g}\right)}^{2}+\frac{W_{h}}{4}{\left(h_{p}-h_{g}\right)}^{2}$$

Accordingly, the squared shape-aware Wasserstein distance is formulated as:

(26) $$W_{Shape}^{2}\left(B_{p},B_{g}\right)=D_{shape}+S_{shape}$$

Unlike conventional NWD, this formulation imposes direction-dependent penalties according to the intrinsic geometry of the ground-truth box. It therefore strengthens geometric constraints for elongated defects while maintaining balanced regression for approximately circular or square targets. Finally, the normalized Shape-NWD similarity and regression loss are calculated as:

(27) $$Shape-NWD\left(B_{p},B_{g}\right)=\exp\left[-\frac{\sqrt{W_{Shape}^{2}\left(B_{p},B_{g}\right)}}{C}\right]\;L_{shape-NWD}=1-shape-NWD\left(B_{p},B_{g}\right)$$

where C is the normalization constant associated with the target scale. Shape-NWD combines distribution-space similarity with shape-conditioned directional modulation, reducing sensitivity to minor coordinate perturbations and providing stable optimization for small, elongated, and weakly overlapping defects. Since it is used only during training, it introduces no additional parameters or inference cost.

Accordingly, Shape-NWD is treated as an established complementary training objective in this study, whereas the methodological novelty of CRF-YOLO is primarily attributed to the proposed feature-transformation, defect-evidence modeling, and discrepancy-guided reconciliation architecture.

## 4. Experiments and Results

### 4.1. Experimental Setup

The experimental dataset was constructed by integrating two publicly available PCB defect datasets. The first source was obtained from the Roboflow Universe platform and contained 2538 annotated images, whereas the second source was the open-source PCB defect dataset released by Peking University, containing 1386 images [2]. Both datasets cover six representative defect categories: missing hole, mouse bite, open circuit, short circuit, spur, and spurious copper. Before merging, the category names were standardized, and all annotations were converted into the YOLO format using normalized center coordinates, width, and height.

A multistage data-quality inspection was then performed. Corrupted or unreadable images, missing annotation files, invalid bounding boxes, and duplicated samples were removed. Following this process, 3847 valid parent images were retained, including 2488 images from the Roboflow dataset and 1359 images from the PKU dataset. To preserve fine-grained features of small PCB defects, deterministic defect-aware cropping and image tiling were applied, and the resulting samples were standardized to 640 × 640 pixels. Although the two datasets adopted different original annotation conventions, defect categories with identical semantic meanings were merged into a unified six-class definition. The bounding-box coordinates were transformed synchronously with the images. This preprocessing procedure generated 15,388 valid detection samples.

All cropping and tiling operations were performed after assigning parent images to the corresponding subsets, ensuring that spatially correlated samples generated from the same original image were restricted to a single subset. To prevent information leakage, dataset partitioning was controlled according to the identifiers of the original parent images. Cropped samples and geometrically related images originating from the same parent image were assigned exclusively to one subset. Source-aware and category-aware stratification was adopted to maintain comparable data distributions across the three subsets. The resulting dataset contained 12,319 training images, 1534 validation images, and 1535 test images, corresponding to an approximate ratio of 8:1:1.

**Figure 5:**
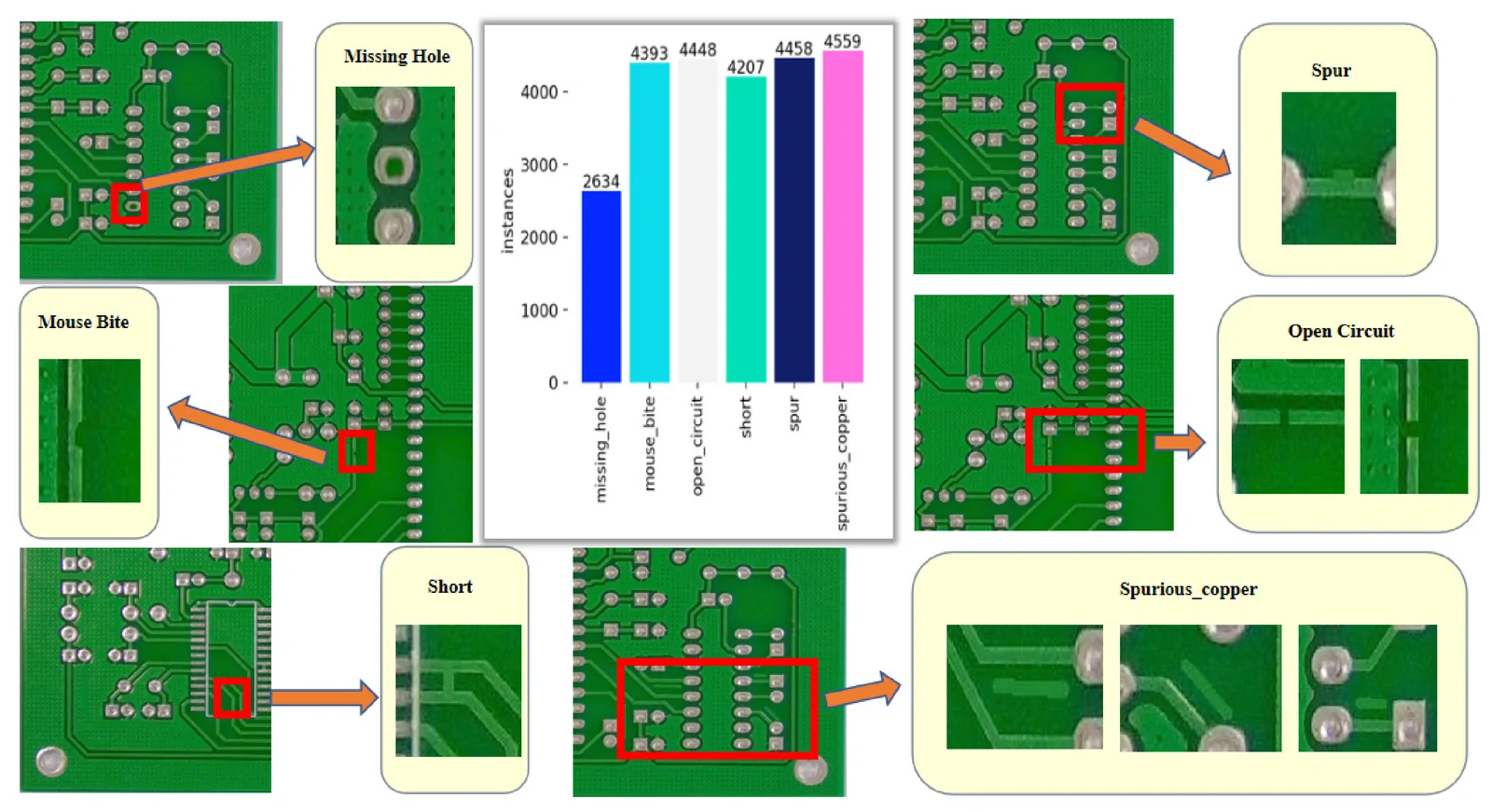
Distribution of annotated defect instances in the training set.

Stochastic data augmentation was applied exclusively to the training subset during batch loading. The augmentation operations included random horizontal flipping, translation, scaling, HSV perturbation, and Mosaic augmentation. Image and bounding-box transformations were performed simultaneously. No stochastic augmentation was applied to the validation or test sets, which underwent only deterministic resizing and padding. Since augmentation was performed online during training rather than generating permanent image files, no additional augmented images were counted in the dataset statistics, the indexed training-set size remained 12,319. The training set contained 2634 missing-hole, 4393 mouse-bite, 4448 open-circuit, 4207 short-circuit, 4458 spur, and 4559 spurious-copper instances, as illustrated in Table 4 and Figure 5.

#### 4.1.1. Implementation Details

The primary ablation experiments and comparisons with mainstream detectors were conducted using a random seed of 0. To assess the sensitivity of the results to stochastic training variation, YOLO11n and CRF-YOLO were additionally trained using random seeds of 42 and 3407. Thus, each of the two key models was independently trained three times using the same dataset partition, optimizer, input resolution, number of training epochs, and remaining hyperparameters, with only the random seed being changed. All trained models were evaluated on the same fixed test set. The repeated-run results are reported as the arithmetic mean ± sample standard deviation over the three independent runs.

All experiments were conducted using the hardware and software environment summarized in Table 5. YOLO11n was adopted as the baseline, and CRF-YOLO was constructed by incorporating C3k2-Lite, RFFA, RCF, and Shape-NWD while retaining the original backbone. The proposed feature transformation and enhancement modules were selectively deployed in the neck, as described in Section 3, whereas Shape-NWD was used only during training and introduced no additional parameters or computational overhead during inference.

All models were trained for 300 epochs with a batch size of 16 and an input image resolution of 640×640. Stochastic gradient descent was used as the optimizer, and the initial learning rate was set to 0.01. Unless otherwise specified, the remaining training parameters followed the default settings of the Ultralytics framework. To ensure a fair comparison, the same dataset partition and training configuration were adopted for the ablation experiments and mainstream detector comparisons.

The main training parameters are summarized in Table 6.

For the controlled comparison with PCB-specific detectors presented in Section 4.4, MS-DETR [36], PD-YOLOv8 [37], YOLOv8-PCB [38], and YOLO-MobileViT [39] were implemented and independently retrained on the unified PCB dataset constructed in this study. The detection heads of all models were configured for the same six defect categories. All compared models used the fixed parent-image-level training, validation, and test partitions described in Section 4.1, comprising 12,319 training images, 1534 validation images, and 1535 test images, respectively. The input resolution, training-only augmentation pipeline, number of training epochs, batch size, random seed, model-selection criterion, and evaluation procedure were kept consistent across the compared models. The best-performing checkpoint on the validation set was selected for each model and subsequently evaluated on the same independent test set. The parameter count and GFLOPs of each detector were recalculated at an input resolution of 640 × 640. Therefore, all numerical results from the PCB-specific model comparison were obtained from experiments conducted in this study rather than extracted from the original publications. References [36,37,38,39] are provided only to identify the original sources of the corresponding model architectures.

#### 4.1.2. Evaluation Metrics

To comprehensively evaluate the detection accuracy and computational efficiency of the proposed model, Precision, Recall, mAP@0.5, mAP@0.5:0.95, the number of parameters, and floating-point operations were selected as evaluation metrics.

Precision measures the proportion of correctly detected defect instances among all instances predicted as defects and is calculated as:

(28) $$P=\frac{TP}{TP+FP}$$

where TP represents the number of correctly detected positive instances and FP denotes the number of background regions or incorrect categories that are falsely predicted as defects.

Recall evaluates the proportion of ground-truth defect instances that are successfully detected and is expressed as:

(29) $$R=\frac{TP}{TP+FN}$$

where FN represents the number of defect instances missed by the detector. A higher Precision indicates fewer false detections, whereas a higher Recall indicates fewer missed detections.

Average Precision is obtained by integrating the precision–recall curve of the i-th defect category:

(30) $${AP}_{i}=\int_{0}^{1}P_{i}(R)dR$$

where $P_{i}(R)$ denotes the Precision corresponding to a given Recall level for category i. Mean Average Precision is calculated by averaging the AP values over all defect categories:

(31) $$mAP=\frac{1}{N_{c}}\sum_{i=1}^{N_{c}}{AP}_{i}$$

where $N_{c}$ denotes the number of defect categories, which is six in this study.

The mAP@0.5 metric represents the mean AP calculated at an IoU threshold of 0.5 and primarily reflects the overall detection and classification ability of the model. In contrast, mAP@0.5:0.95 averages the AP values over IoU thresholds ranging from 0.50 to 0.95 with an interval of 0.05. It imposes stricter requirements on bounding-box localization and is therefore more suitable for evaluating the localization accuracy of small PCB defects.

In addition to detection accuracy, Params and GFLOPs were adopted to evaluate model complexity. Params represents the total number of trainable parameters and reflects the model storage requirement, whereas GFLOPs measures the number of floating-point operations required for a single forward pass. Lower Params and GFLOPs generally indicate a more lightweight architecture and lower computational cost. Therefore, the selected metrics jointly evaluate CRF-YOLO in terms of classification accuracy, detection completeness, localization precision, and deployment efficiency.

Frames per second (FPS) was employed to evaluate the actual inference efficiency of each detector. FPS is calculated as (FPS = N/T), where (N) denotes the number of processed images and (T) represents the synchronized model-forward execution time in seconds. Params and GFLOPs characterize the theoretical storage and computational complexity of a model, whereas FPS reflects its actual execution efficiency on the specified hardware. The measured throughput is also affected by operator organization, memory access, GPU parallelism, and CUDA kernel scheduling. A higher FPS indicates stronger real-time inference capability under the adopted experimental environment.

### 4.2. Ablation Experiments

To evaluate the effectiveness of the proposed components, a series of ablation experiments was conducted using YOLO11n as the baseline. All models were trained and evaluated using the same dataset partition, input resolution, training epochs, optimizer, and hyperparameter settings.

#### 4.2.1. Overall Ablation Study

For the RCF-only configuration (No. 4), two identical raw P3 features were used as the inputs of RCF, enabling its independent contribution to be evaluated while preserving its dual-input structure.

The overall ablation experiments were conducted by separately and progressively introducing the proposed C3k2-Lite, RFFA, and RCF modules, together with the existing Shape-NWD regression loss [22], into YOLO11n. The results are presented in Table 7.

As shown in Table 7, the four components exhibit distinct yet complementary effects. C3k2-Lite reduces Params from 2.583 M to 2.138 M and GFLOPs from 6.3 to 5.6, while improving mAP@0.5 and mAP@0.5:0.95 by 0.32 and 1.03 percentage points, respectively. This result validates its efficient partial-channel feature transformation, which removes redundant computation without sacrificing detection capability. RFFA provides the largest standalone gain in mAP@0.5, increasing it by 1.24 percentage points, together with a 0.79-point improvement in Precision. Its heterogeneous defect-evidence modeling therefore enhances foreground discrimination, although the slight decrease in Recall indicates that selective recalibration may suppress several weak defect responses. In contrast, the controlled RCF-without-RFFA configuration increases Recall by 0.69 percentage points and mAP@0.5:0.95 by 1.04 percentage points, indicating that the structure-aware reconstruction within RCF retains an independent effect even without the RFFA-enhanced input. Shape-NWD further improves the two mAP metrics by 0.63 and 1.12 percentage points without increasing inference complexity, confirming the benefit of shape-sensitive distributional regression for small and irregular defects.

The progressive results reveal that these modules are not simply additive but function through coordinated compensation. Combining C3k2-Lite and RFFA retains a lightweight configuration and achieves 154.48 FPS, although the interaction between feature compression and selective evidence recalibration slightly limits the expected accuracy gain. After RCF is introduced, Precision, Recall, mAP@0.5, and mAP@0.5:0.95 increase to 96.24%, 89.85%, 94.21%, and 51.46%, respectively. Meanwhile, the model maintains 157.63 FPS with only 0.016 M additional parameters and 0.2 GFLOPs. This recovery indicates that RCF effectively reconciles the information-preserving raw stream with the discriminative RFFA stream.

Finally, incorporating Shape-NWD raises Recall by 1.41 percentage points and further improves both mAP metrics, producing the complete CRF-YOLO with 95.53% Precision, 91.26% Recall, 94.46% mAP@0.5, and 51.74% mAP@0.5:0.95. Compared with YOLO11n, the final model improves these four metrics by 1.02, 1.83, 1.81, and 2.03 percentage points, respectively, while reducing Params by approximately 15.8% and GFLOPs by 4.8%. Its inference speed reaches 157.15 FPS, representing an increase of 14.72% over the baseline. These results demonstrate a clear division of functionality: C3k2-Lite establishes computational efficiency, RFFA strengthens defect discrimination, RCF restores and reconciles weak structural evidence, and Shape-NWD stabilizes geometric regression. Their coordinated interaction enables CRF-YOLO to achieve a favorable balance between detection accuracy and model complexity.

#### 4.2.2. Comparison of Lightweight Modules

To verify the efficiency of C3k2-Lite, it was compared with the original C3k2 module, the Ghost-convolution-based C3Ghost structure [14], and C2fCIB [39] derived from the compact inverted block design of YOLOv10. All modules were evaluated at the same neck positions under identical training settings, and the results are presented in Table 8.

C2fCIB obtains the highest mAP@0.5 among the compared modules, reaching 93.01%, while C3Ghost achieves 93.00%. C3k2-Lite obtains a closely comparable mAP@0.5 of 92.97%, only 0.04 percentage points lower than C2fCIB and 0.03 percentage points lower than C3Ghost.

In terms of complexity, C3k2-Lite achieves the lowest parameter count and computational cost. Compared with the original C3k2, it reduces the parameter count by 0.445 M and GFLOPs by 0.7 while increasing mAP@0.5 by 0.32 percentage points. Compared with C3Ghost, C3k2-Lite reduces the parameter count by a further 0.166 M and GFLOPs by 0.4, with only a negligible 0.03-percentage-point reduction in mAP@0.5. Compared with C2fCIB, it reduces Params by 0.309 M and GFLOPs by 0.7.

These results indicate that the marginal accuracy advantage of C3Ghost and C2fCIB is achieved at a noticeably higher computational cost. In contrast, C3k2-Lite provides a more favorable balance between detection accuracy and efficiency. Therefore, C3k2-Lite was selected as the lightweight feature extraction module of CRF-YOLO.

#### 4.2.3. Comparison of Attention Modules

To demonstrate the effectiveness of RFFA, it was compared with four commonly used attention mechanisms: squeeze-and-excitation attention, convolutional block attention, coordinate attention, and efficient channel attention. Each attention module was independently introduced into the same YOLO11n baseline under identical experimental settings. The results are shown in Table 9.

All attention mechanisms improve mAP@0.5 relative to the baseline, confirming that feature recalibration is beneficial for PCB defect detection. SE increases mAP@0.5 by 0.33 percentage points, whereas ECA improves it by 0.60 percentage points without increasing the parameter count. CBAM and CA achieve stronger gains of 0.68 and 0.74 percentage points, respectively, indicating that jointly or directionally modeling spatial information is more effective than relying solely on channel recalibration.

RFFA achieves the best result, increasing mAP@0.5 from 92.65% to 93.89%, corresponding to an improvement of 1.24 percentage points. Its mAP@0.5 is 0.50 percentage points higher than that of CA, which is the second-best attention mechanism. This advantage can be attributed to the defect-oriented feature representation of RFFA. Instead of applying a generic channel or spatial weighting operation, RFFA separately models position, edge, connectivity, and texture information and adaptively fuses these responses. These feature types correspond closely to the visual characteristics of the six PCB defect categories.

The improvement requires only an additional 0.021 M parameters and 0.2 GFLOPs. Although its complexity is slightly higher than that of conventional lightweight attention mechanisms, the additional cost remains small relative to its accuracy gain. The comparison therefore supports the use of RFFA as the principal feature enhancement module in CRF-YOLO.

#### 4.2.4. Analysis of the RCF Module

Although the progressive ablation study in Table 7 verifies the effectiveness of the complete module sequence, it does not fully separate the independent effect of RCF from its interaction with RFFA. A controlled interaction experiment was therefore conducted with C3k2-Lite fixed as the common lightweight configuration. RFFA and RCF were introduced separately and jointly to distinguish their individual contributions from their coupled effect. Because the standard RCF receives both the raw P3 feature and its RFFA-enhanced counterpart, the RCF-without-RFFA configuration used two identical raw P3 features as inputs. This controlled setting retains the input dimensions and internal processing of RCF while removing the enhancement supplied by RFFA. The results are presented in Table 10.

As shown in Table 10, RFFA increases mAP@0.5 from 92.97% to 93.19% under the fixed C3k2-Lite configuration, corresponding to an independent improvement of 0.22 percentage points. The controlled RCF-without-RFFA configuration achieves 93.62% mAP@0.5, providing an improvement of 0.65 percentage points. These results indicate that RFFA contributes defect-oriented feature enhancement, whereas the detail-reconstruction and position-aware recalibration operations within RCF retain an independent effect even when no RFFA-enhanced representation is provided.

Jointly incorporating RFFA and RCF further increases mAP@0.5 to 94.21%, outperforming the RFFA-only and controlled RCF-without-RFFA configurations by 1.02 and 0.59 percentage points, respectively. Relative to C3k2-Lite, the joint improvement is 1.24 percentage points, whereas the sum of the two independent improvements is 0.87 percentage points. The additional margin of 0.37 percentage points provides evidence of a positive non-additive interaction under the adopted configuration. RFFA strengthens discriminative defect responses, while RCF uses the discrepancy between the raw and enhanced representations to recover weak structural information and control feature injection. Their joint operation therefore produces a more effective representation than either module alone, with a limited complexity of 2.175 M parameters and 6.0 GFLOPs.

**Figure 6:**
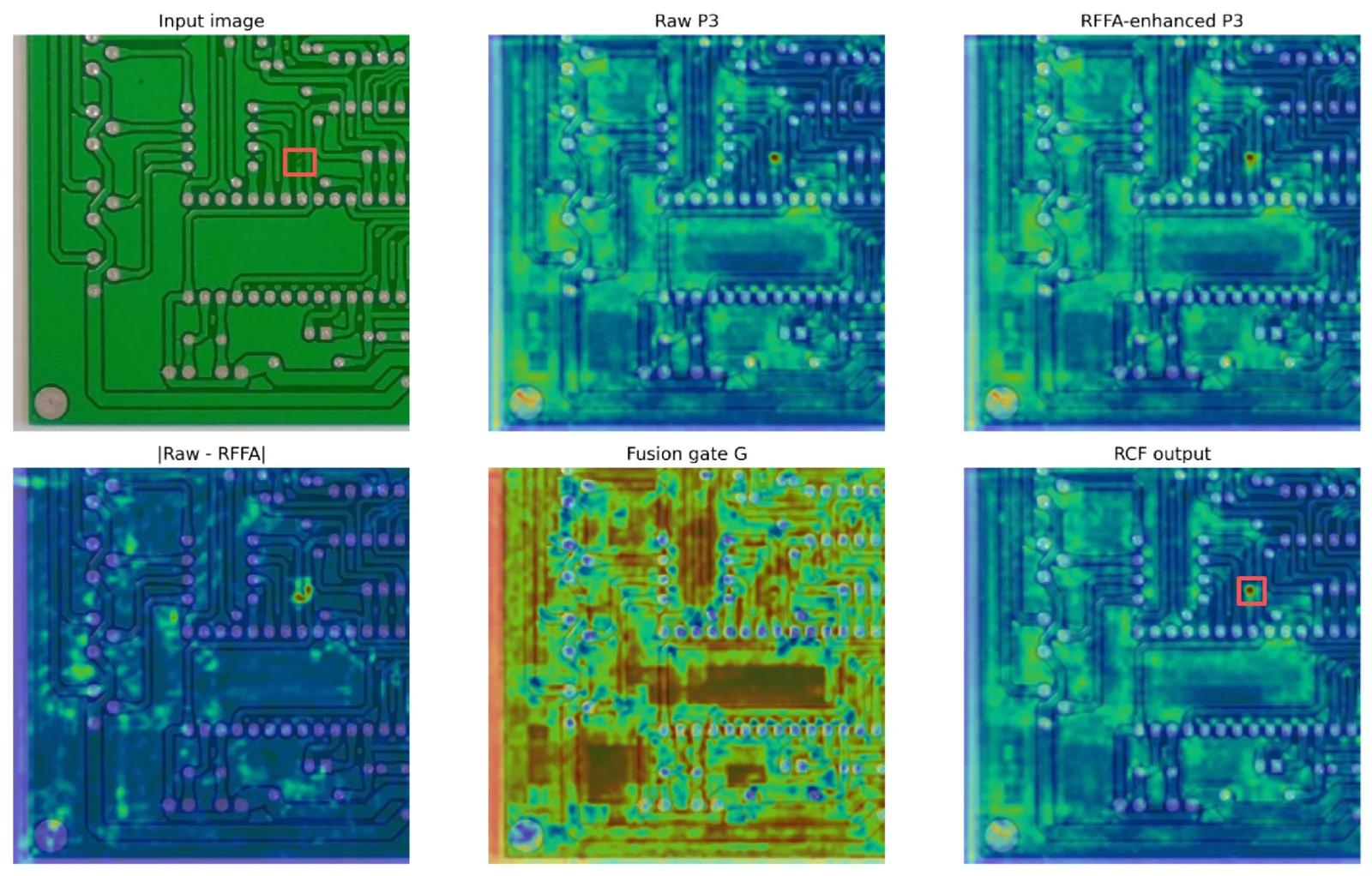
Visualization of RCF feature responses. The red boxes indicate the annotated open-circuit defect region in the input image and the corresponding response region in the RCF output.

To examine the mechanism underlying the positive interaction observed in Table 10, intermediate feature responses were extracted from the same open-circuit sample under an identical visualization protocol. Figure 6 presents the raw P3 response, the RFFA-enhanced response, their cross-stream discrepancy, the learned fusion gate, and the final RCF output, thereby providing a qualitative view of how enhancement and information recovery are coordinated.

The raw P3 feature preserves the overall topology of conductive traces and solder pads, but its activation remains widely distributed over structurally similar background regions. After RFFA processing, the global circuit structure is retained while the response around the open-circuit defect becomes more discriminative. The discrepancy map $|F_{raw}-F_{RFFA}|$ further isolates the localized feature variation introduced by RFFA, producing a compact high-response region near the defect location. Based on the raw feature, enhanced feature, and their discrepancy, the fusion gate G performs location-adaptive feature arbitration. It should be noted that G is not a direct defect-localization map; instead, it determines the relative contribution of the two streams at different spatial positions. The resulting RCF output preserves the information-rich structural context of the raw stream while maintaining the discriminative response supplied by RFFA. These observations indicate that RCF performs discrepancy-guided dual-stream feature reconciliation rather than simple feature accumulation, thereby compensating for weak structural evidence that may be attenuated during selective feature recalibration. This qualitative behavior is consistent with the improvements in Recall and mAP@0.5:0.95 observed after introducing RCF in the overall ablation study. Together, the controlled interaction ablation and feature-response visualization demonstrate that RFFA and RCF perform complementary enhancement and information-recovery functions rather than acting as independently stacked modules.

#### 4.2.5. Comparison of Bounding-Box Regression Losses

To evaluate the effectiveness of Shape-NWD, five bounding-box regression losses (CIoU [29], UIoU [21], MPDIoU [20], NWD [7], and Shape-NWD [22]) were compared under the same YOLO11n architecture and training configuration. Because the loss function affects only the training objective, all models retain the same parameter count and computational complexity. The results are listed in Table 11.

CIoU, which is used as the baseline regression loss, obtains an mAP@0.5 of 92.65%. UIoU produces only a limited improvement of 0.05 percentage points, while MPDIoU increases mAP@0.5 to 92.95%. NWD achieves a stronger result of 93.11%, indicating that distribution-based box similarity is more suitable than overlap-dominated regression for the small PCB defects in the dataset.

Shape-NWD achieves the highest mAP@0.5 of 93.28%, improving upon CIoU, MPDIoU, UIoU, and NWD by 0.63, 0.33, 0.58, and 0.17 percentage points, respectively. Compared with conventional NWD, Shape-NWD introduces shape-aware directional constraints that distinguish localization errors along the long and short sides of a bounding box. This characteristic is particularly relevant to elongated open-circuit, short-circuit, and spur defects, for which identical coordinate deviations in different directions may produce different localization consequences.

No additional parameters or GFLOPs are introduced by Shape-NWD. Therefore, its performance improvement originates entirely from a more appropriate regression objective rather than an increase in model capacity. The results verify that Shape-NWD provides a more effective localization constraint for small and morphologically diverse PCB defects and is suitable for integration into the final CRF-YOLO model.

### 4.3. Comparison with Mainstream Object Detectors

To evaluate the overall competitiveness of CRF-YOLO, it was compared with representative YOLO-series detectors under the same dataset partition and training configuration. The results are shown in Table 12.

CRF-YOLO achieves the highest Precision, Recall, mAP@0.5, and mAP@0.5:0.95 among the compared models. Compared with the YOLO11n baseline, its Precision and Recall increase by 1.02 and 1.83 percentage points, respectively, indicating that the proposed model simultaneously reduces false detections and missed defects. Its mAP@0.5 and mAP@0.5:0.95 increase by 1.81 and 2.03 percentage points, demonstrating improved overall detection and more accurate bounding-box localization. Meanwhile, the parameter count decreases from 2.583 M to 2.175 M, and GFLOPs decrease from 6.3 to 6.0. The measured inference speed increases from 136.99 FPS to 157.15 FPS, providing further evidence that the reduction in model complexity is translated into practical execution efficiency.

YOLO26 provides the second-best overall accuracy, but CRF-YOLO still improves Recall by 2.49 percentage points, mAP@0.5 by 1.41 percentage points, and mAP@0.5:0.95 by 1.34 percentage points. CRF-YOLO also exceeds the 148.37 FPS of YOLO26 by 8.78 FPS while using fewer parameters. Although YOLOv9t has a slightly smaller parameter count, its Recall, two mAP metrics, and inference speed are all lower than those of CRF-YOLO.

These results indicate that the proposed model achieves a better balance between accuracy and complexity. C3k2-Lite reduces redundant computation, RFFA and RCF strengthen small-defect feature representation and fusion, and Shape-NWD improves bounding-box localization. Their complementary effects enable CRF-YOLO to outperform general-purpose detectors on the PCB defect dataset.

#### Robustness Across Different Random Seeds

To complement the seed-0 comparison reported in Table 12 and determine whether the observed performance improvement is sensitive to stochastic training variation, YOLO11n and CRF-YOLO were independently trained using random seeds of 0, 42, and 3407. The dataset partition and all remaining training configurations were kept unchanged, and every trained model was evaluated on the same fixed test set. The results are reported in Table 13 as individual runs and the corresponding mean ± standard deviation.

As shown in Table 13, CRF-YOLO consistently outperforms YOLO11n under all three random seeds. Averaged over the three independent runs, CRF-YOLO achieves a Precision of 95.54%, a Recall of 91.26%, an mAP@0.5 of 94.47%, and an mAP@0.5:0.95 of 51.74%. Compared with YOLO11n, the corresponding mean values increase by 1.04, 1.86, 1.84, and 2.04 percentage points, respectively. The consistent improvements in both Precision and Recall indicate that CRF-YOLO reduces false detections and missed defects across different training initializations, while the gains in the two mAP metrics confirm more reliable overall detection and bounding-box localization.

The standard deviations of the four evaluation metrics range from 0.17 to 0.19 percentage points for CRF-YOLO, demonstrating limited sensitivity to random-seed variation. In addition, the standard deviations of CRF-YOLO are comparable to or lower than those of YOLO11n, while its performance remains consistently higher in every repeated run. These findings demonstrate that the improvements introduced by CRF-YOLO are reproducible and are not attributable to a single favorable initialization. The results in Table 12 are retained from seed 0 to ensure identical experimental conditions for the comparison with mainstream detectors, whereas Table 13 provides an independent assessment of the robustness of YOLO11n and CRF-YOLO across different random seeds.

### 4.4. Comparison with PCB-Specific Defect Detection Models

Although the preceding experiments demonstrate the effectiveness of CRF-YOLO through comparisons with general-purpose object detectors, PCB surface defects have distinct visual characteristics, including small spatial scales, weak texture responses, blurred boundaries, and high similarity between different defect categories. General-purpose detectors are not explicitly designed to address these task-specific challenges. Therefore, a controlled comparison with representative PCB-specific defect detection models was conducted to further evaluate the accuracy–complexity trade-off of CRF-YOLO.

Four recently proposed PCB-specific detectors were included in the comparison: MS-DETR [40], PD-YOLOv8 [36], YOLOv8-PCB [37], and YOLO-MobileViT [38]. These models represent different technical routes for PCB defect detection. MS-DETR combines multi-stage convolution, scale-adaptive feature fusion, and attention-based global modeling to enhance multi-scale defect representation. PD-YOLOv8 employs a dedicated small-object detection head and efficient multi-scale feature fusion to retain high-resolution spatial information associated with minute defects. YOLOv8-PCB integrates lightweight spatial–channel attention, IdentityFormer-based feature interaction, and an improved bounding-box regression strategy. YOLO-MobileViT combines lightweight Transformer modeling with convolutional feature extraction to reduce the storage requirements of PCB defect detectors.

To ensure experimental comparability, the four PCB-specific detectors were independently retrained using the unified dataset and controlled experimental protocol adopted in this study. All models used the same fixed parent-image-level data partition, six-class annotation definition, input resolution, training-only augmentation pipeline, training budget, model-selection criterion, and test set. Their detection performance was calculated using the same evaluation implementation, while their parameter counts and GFLOPs were recalculated under the same 640 × 640 input setting. Consequently, the values reported in Table 14 are results obtained from the experiments conducted by the authors in this study and are not values transferred from Refs. [36,37,38,39].

As shown in Table 14, all five detectors were trained and evaluated using the same dataset and controlled evaluation protocol. Under these unified conditions, MS-DETR achieves an mAP@0.5 of 86.70%, with 14.000 M parameters and 45.6 GFLOPs. Its relatively high computational cost is associated with its multi-scale Transformer architecture and global feature-interaction operations, which increase the resources required for inference.

PD-YOLOv8 reduces the model complexity to 5.200 M parameters and 10.3 GFLOPs by incorporating a small-object detection branch and multi-scale feature fusion. It achieves an mAP@0.5 of 84.10% on the unified test set. Although its high-resolution detection branch increases the sensitivity to small objects, the result indicates that further feature discrimination is required for PCB defects with similar textures and geometric appearances.

Among the four reproduced PCB-specific detectors, YOLOv8-PCB achieves the highest mAP@0.5 of 90.60%, with 2.500 M parameters and 7.1 GFLOPs. Its lightweight attention and feature-interaction mechanisms provide a comparatively favorable balance between defect representation and model complexity. YOLO-MobileViT further reduces the parameter count to 1.800 M, making it the smallest detector in Table 14. However, it obtains an mAP@0.5 of 87.90% and requires 8.2 GFLOPs, indicating that reducing the parameter count alone does not necessarily result in lower computational cost or stronger defect representation.

Under the same training and evaluation conditions, CRF-YOLO achieves an mAP@0.5 of 94.46% with 2.175 M parameters and 6.0 GFLOPs. Compared with YOLOv8-PCB, which is the second-best model in terms of mAP@0.5, CRF-YOLO improves mAP@0.5 by 3.86 percentage points while reducing the parameter count and GFLOPs by 13.0% and 15.5%, respectively. Compared with YOLO-MobileViT, CRF-YOLO requires only 0.375 M additional parameters but improves mAP@0.5 by 6.56 percentage points and reduces GFLOPs by approximately 26.8%. Consequently, CRF-YOLO achieves the highest mAP@0.5 and the lowest GFLOPs while maintaining the second-smallest parameter count among the five detectors evaluated under the controlled experimental protocol.

The favorable accuracy–complexity balance of CRF-YOLO is achieved without relying on a computationally intensive Transformer backbone or an additional high-resolution detection branch. C3k2-Lite reduces redundant spatial computation, RFFA strengthens the representation of weak defect evidence, and RCF selectively reconciles the raw and attention-enhanced features while recovering structural details. The controlled comparison therefore provides direct experimental evidence that CRF-YOLO combines competitive PCB defect-detection accuracy with lightweight computational characteristics.

### 4.5. Performance Analysis

The class-wise performance of CRF-YOLO on the independent test set is presented in Table 15. The test set contains 1535 images and 3005 defect instances.

CRF-YOLO achieves an AP@0.5 above 90% for all six categories, demonstrating stable multi-class detection performance. Missing hole obtains the best result, with a Recall of 100.0% and an AP@0.5 of 99.4%, owing to its clear contour and relatively consistent morphology. Short and spurious copper also achieve strong detection performance, with AP@0.5 values of 96.8% and 93.4%, respectively. In contrast, mouse bite, open circuit, and spur are more challenging because of their small scales, irregular boundaries, and weak contrast with surrounding conductive patterns. In particular, mouse bite yields the lowest Recall of 85.7%, while open circuit obtains the lowest AP@0.5:0.95 of 44.4%, indicating that precise localization of narrow discontinuities remains difficult.

**Figure 7:**
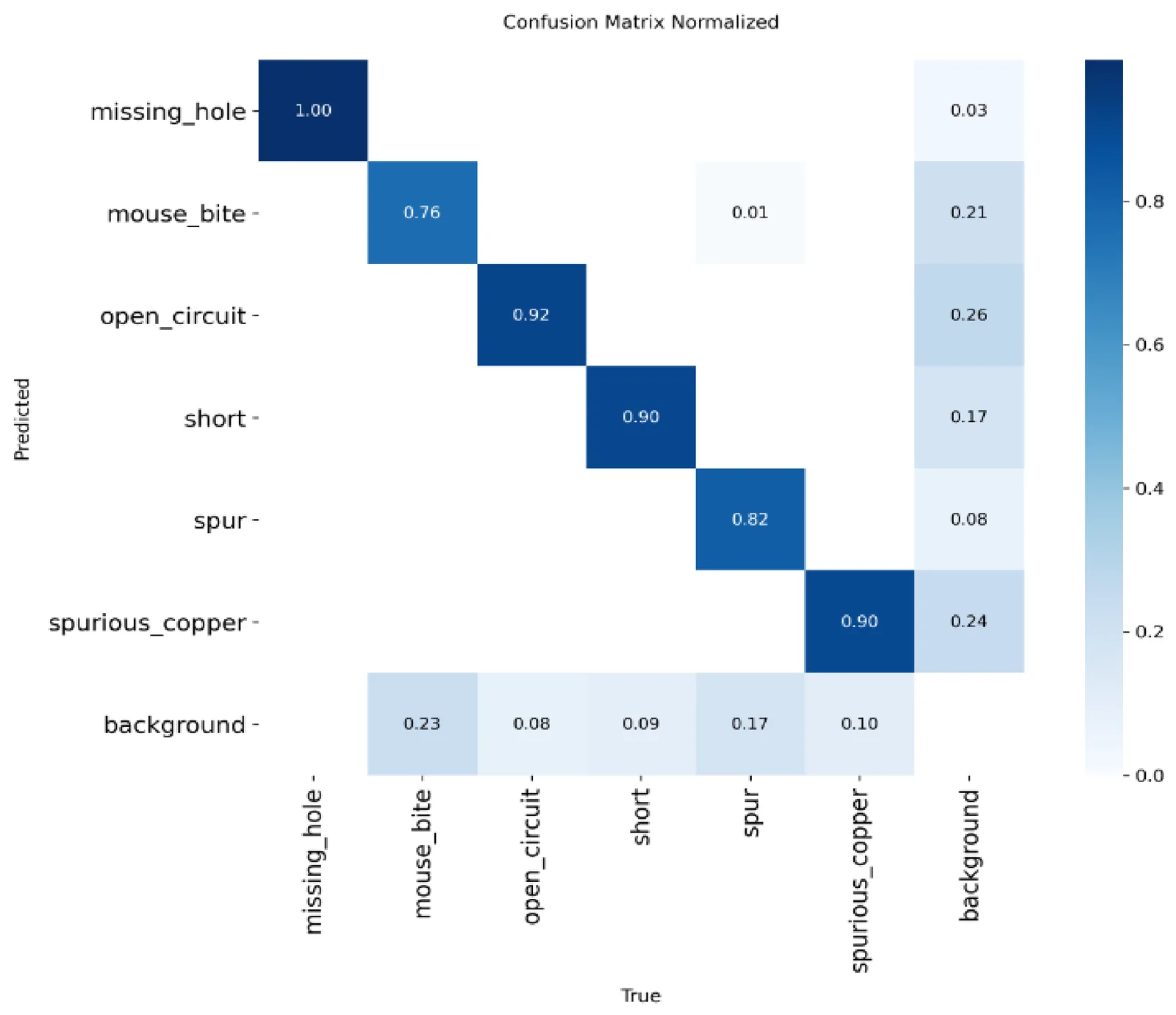
Normalized confusion matrix of CRF-YOLO.

The normalized confusion matrix in Figure 7 further illustrates the class-discrimination capability of CRF-YOLO. Most responses are concentrated along the diagonal, indicating limited confusion among the six defect categories. Missing hole and short exhibit particularly reliable classification, whereas the remaining errors mainly occur between defects with similar local geometric patterns or weak structural differences. These results are consistent with the class-wise statistics in Table 15.

**Figure 8:**
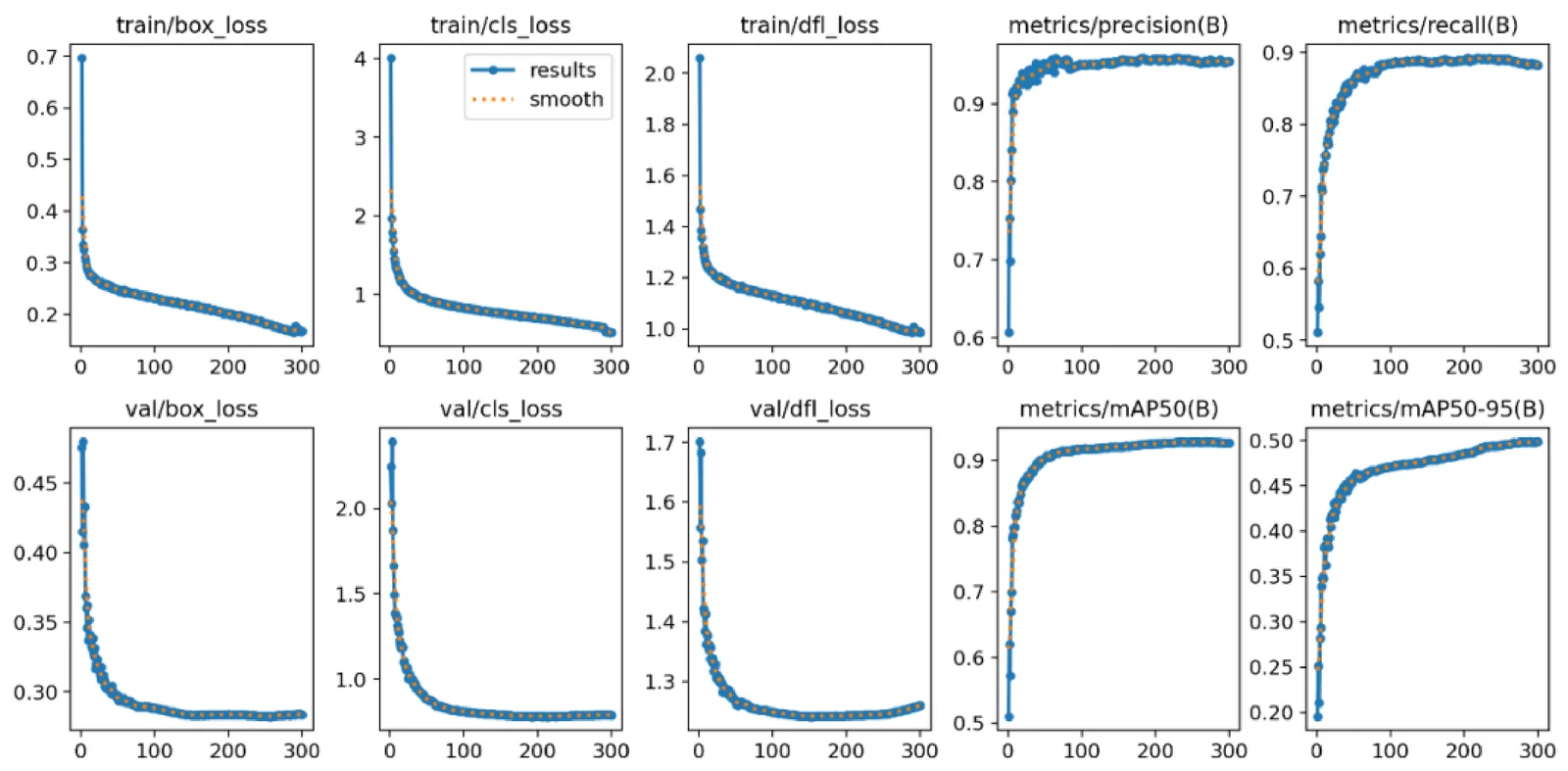
Training and validation curves of CRF-YOLO. The legend applies to all subfigures.

The training and validation curves of CRF-YOLO are shown in Figure 8. The box, classification, and distribution focal losses decrease rapidly during the early training stage and then converge gradually, while the corresponding validation losses follow similar trends. Precision, Recall, mAP@0.5, and mAP@0.5:0.95 increase steadily and stabilize in the later epochs. No evident divergence is observed between the training and validation curves, indicating stable optimization and satisfactory generalization under the adopted training protocol.

**Figure 9:**
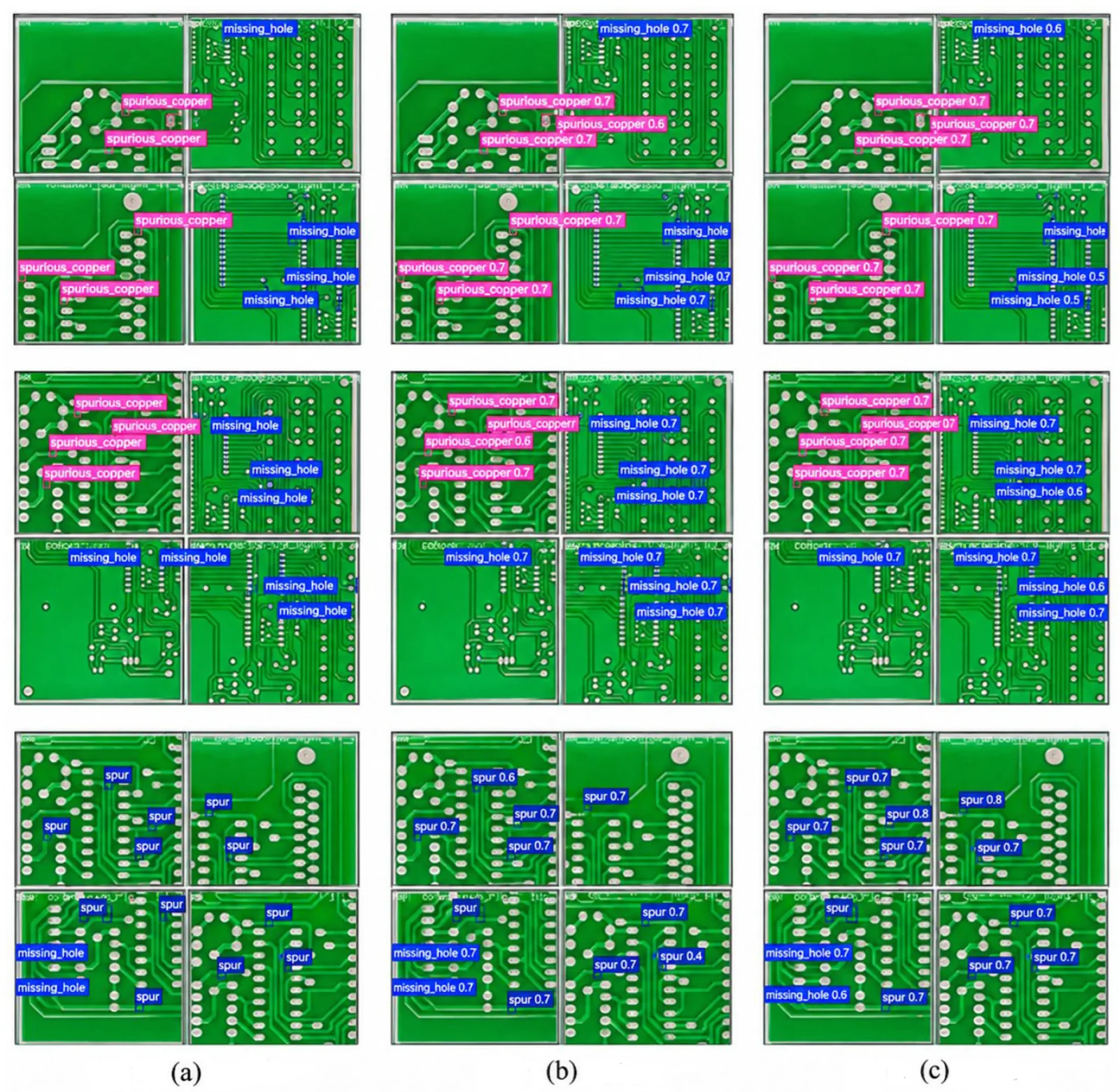
Visual comparison of ground-truth annotations, YOLO11n predictions, and CRF-YOLO predictions on representative test samples: (**a**) ground truth; (**b**) YOLO11n; and (**c**) CRF-YOLO.

Representative detection results are presented in Figure 9. The selected samples contain missing-hole, spurious-copper, and spur defects under dense circuit layouts and different spatial distributions. CRF-YOLO accurately identifies most defect instances and produces bounding boxes that closely match their actual locations. It maintains reliable responses for multiple adjacent defects and preserves relatively high confidence for small and irregular targets. These qualitative results support the quantitative findings in Table 12 and Table 15 and demonstrate that the coordinated design of RFFA, RCF, and Shape-NWD improves defect discrimination and localization under complex PCB backgrounds.

Although CRF-YOLO improves the overall detection performance, mouse-bite and open-circuit defects remain relatively difficult because of their weak boundaries and narrow structures. This is consistent with the lower Recall of mouse bite and the lower AP@0.5:0.95 of open circuit. Further improvement may require higher-resolution feature maps or more targeted hard-sample augmentation.

### 4.6. Lightweight Deployment of CRF-YOLO

In addition to detection accuracy, model complexity and deployment convenience are important considerations for practical PCB inspection. The original YOLO11n contains 2.583 M parameters and requires 6.3 GFLOPs, whereas CRF-YOLO contains 2.175 M parameters and requires 6.0 GFLOPs. Therefore, the proposed model reduces the parameter count by approximately 15.8% and the computational cost by approximately 4.8%. Meanwhile, its mAP@0.5 and mAP@0.5:0.95 increase by 1.81 and 2.03 percentage points, respectively. The reduction in complexity is mainly attributed to C3k2-Lite, which offsets the additional computation introduced by RFFA and RCF. Moreover, Shape-NWD is used only during training and introduces no additional parameters or inference cost. Consistent with these reductions, the measured inference speed increases from 136.99 FPS for YOLO11n to 157.15 FPS for CRF-YOLO under the same RTX 3090 and PyTorch environment, further supporting its practical deployment efficiency. These characteristics provide a suitable foundation for lightweight application deployment.

**Figure 10:**
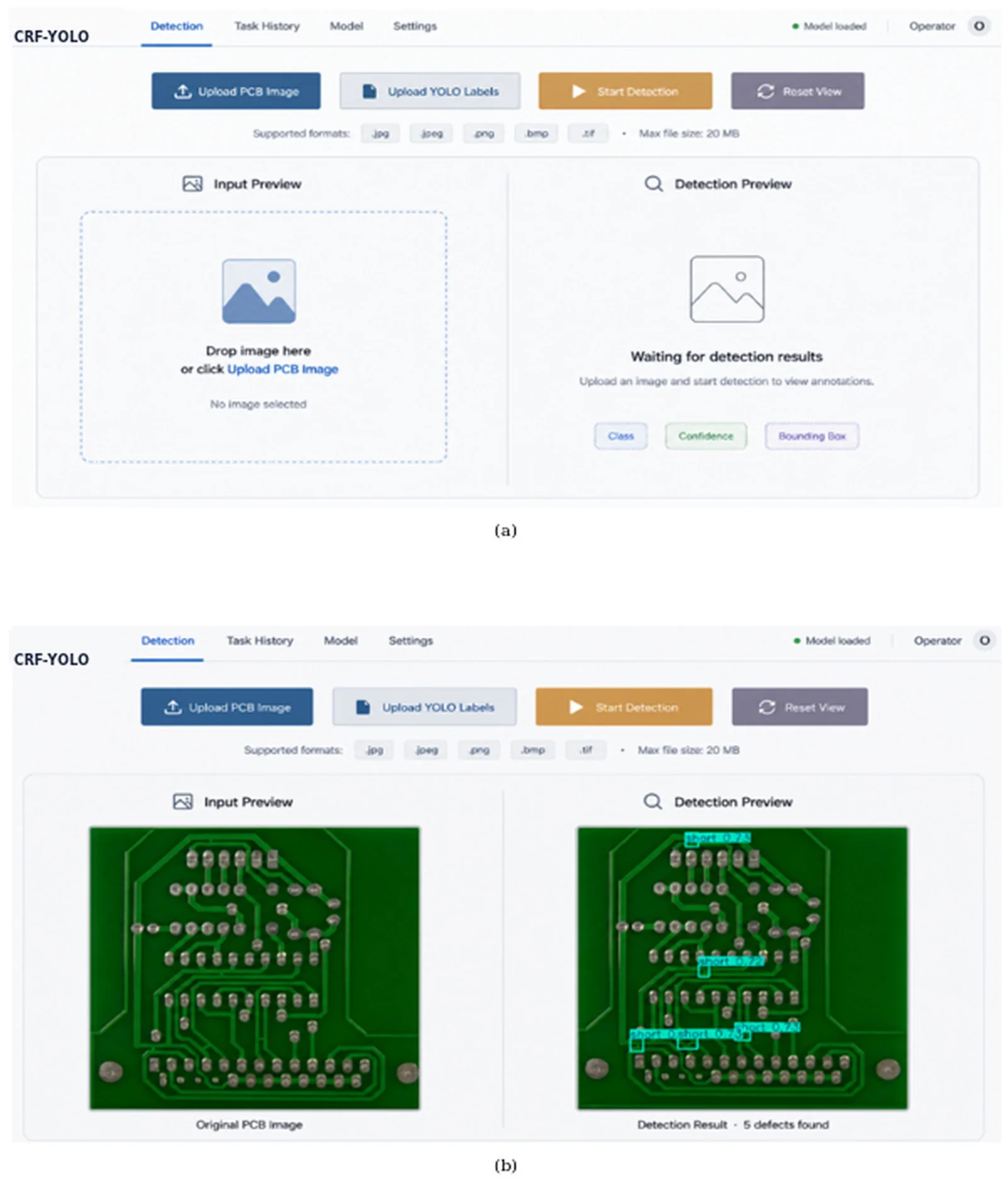
Web-based lightweight deployment interface of CRF-YOLO: (**a**) PCB image upload and detection-control interface; (**b**) visualization of the original PCB image and detection results.

To verify the practical applicability of CRF-YOLO, the trained model was integrated into a browser-based interactive PCB defect detection system. As shown in Figure 10a, the detection interface provides functions for uploading PCB images, importing optional YOLO-format labels, starting the detection process, and resetting the current task. Common image formats, including JPG, JPEG, PNG, BMP, and TIF, are supported. The model-loading status is displayed in the navigation bar, allowing users to confirm that the inference model is available before performing detection. The interface adopts a dual-panel layout, in which the uploaded PCB image and the detection result are presented separately for intuitive comparison.

After a PCB image is uploaded, the system transmits the image to the inference module and performs the required preprocessing before invoking CRF-YOLO. The model subsequently outputs the predicted defect category, confidence score, and bounding-box coordinates. These results are rendered on the original image and returned to the detection-preview panel. As illustrated in Figure 10b, the left panel displays the original PCB image, whereas the right panel presents the annotated detection result. In the demonstrated case, the system identifies five short-circuit defects and marks their locations using bounding boxes together with the corresponding category labels and confidence scores.

The Web-based implementation establishes a complete operational workflow from image input and model invocation to defect localization and result visualization. Compared with directly executing model-inference scripts, the interactive interface reduces the technical requirements for operators and improves the accessibility of the proposed method. More importantly, the successful integration of CRF-YOLO into the detection platform demonstrates that the lightweight improvements do not merely reduce theoretical model complexity, but also support practical PCB defect-inspection applications. The system can therefore provide an accessible auxiliary tool for PCB quality inspection and a foundation for subsequent integration into industrial inspection software.

## 5. Conclusions

This study addresses the weak feature representation, background interference, localization instability, and deployment constraints associated with small PCB defects. Accordingly, a lightweight detection model named CRF-YOLO is developed based on YOLO11n through coordinated optimization of feature extraction, defect-oriented enhancement, feature reconciliation, and bounding-box regression.

C3k2-Lite retains the cross-stage aggregation structure of C3k2 and replaces its internal bottleneck with FasterLiteBlock. By combining partial-channel convolution, bypass transmission, pointwise channel mixing, and scaled residual learning, it reduces redundant spatial computation while preserving fine-grained defect information. RFFA further models four complementary types of defect evidence, namely position, boundary, connectivity, and texture. Its independent branch gating and spatial–channel recalibration enhance weak contour breaks and abnormal conductive structures in repetitive PCB backgrounds. To avoid the loss of low-response cues caused by attention recalibration, RCF adaptively reconciles the raw P3 feature and the RFFA-enhanced feature according to their response discrepancy. It also reconstructs boundary, directional-continuity, and local-contrast information, thereby preserving subtle defect signals while maintaining stable multi-scale feature propagation. Shape-NWD is additionally adopted to improve the localization stability of small, elongated, and irregular defects without increasing inference complexity.

The controlled interaction ablation further shows that the joint RFFA–RCF configuration provides a 1.24-percentage-point improvement in mAP@0.5 over C3k2-Lite, exceeding the summed independent improvements by 0.37 percentage points. Together with the feature-response visualization, this result supports the complementary relationship between defect-evidence enhancement and discrepancy-guided information recovery.

Experimental results demonstrate the effectiveness of CRF-YOLO. The model achieves a Precision of 95.53%, a Recall of 91.26%, an mAP@0.5 of 94.46%, and an mAP@0.5:0.95 of 51.74%. Compared with YOLO11n, these metrics increase by 1.02, 1.83, 1.81, and 2.03 percentage points, respectively, while the parameter count decreases from 2.583 M to 2.175 M and GFLOPs decrease from 6.3 to 6.0. In comparisons with mainstream detectors, CRF-YOLO achieves the best overall accuracy and outperforms the second-best YOLO26 in Recall, mAP@0.5, and mAP@0.5:0.95 while using fewer parameters. In addition, all six defect categories achieve AP@0.5 values above 90%, confirming stable multi-class detection performance and a favorable balance between accuracy and model complexity.

For practical application, CRF-YOLO is integrated into a browser-based PCB defect detection system. The system supports image uploading, optional import of YOLO-format labels, model-status display, detection execution, result resetting, and visual comparison between original and annotated images. It automatically returns defect categories, confidence scores, and bounding-box coordinates, demonstrating the engineering feasibility and operational accessibility of the lightweight model.

Despite the encouraging results, several limitations of the present study should be acknowledged. First, the current evaluation focuses on six common PCB defect categories represented in the integrated public datasets. Although the parent-image-level partitioning and multi-seed experiments provide a reliable assessment under the adopted protocol, evaluations using additional industrial datasets with more diverse PCB layouts, imaging devices, illumination conditions, and production environments would further substantiate the practical adaptability of CRF-YOLO. Second, the efficiency evaluation was performed under a unified PyTorch and GPU configuration to ensure a consistent comparison among the tested models. Complementary experiments on different edge-computing platforms could provide a more comprehensive characterization of its hardware adaptability and deployment efficiency. In addition, the present study mainly addresses image-based defect localization, whereas production-process information and historical inspection knowledge may provide useful contextual support for more comprehensive industrial inspection.

Future work will therefore extend the evaluation of CRF-YOLO to more diverse industrial scenarios, further improve feature representation for weak-edge and narrow defects, and optimize inference performance across heterogeneous edge devices. The integration of CRF-YOLO into a multimodal PCB inspection agent will also be explored, enabling the detector to combine visual results with manufacturing knowledge for defect-risk interpretation, inspection decision support, and automated report generation.

## Figures and Tables

**Table 1:** Functional composition of FasterLiteBlock.

Component	Operation	Functional Role
Channel partition	Partial channel split	Allocates spatial computation only to informative channel subsets
Spatial operator	(3 × 3) Partial Convolution	Extracts local patterns with reduced redundant computation
Bypass pathway	Identity transmission	Preserves low-amplitude and fine-grained defect responses
Channel mixer	Two (1 × 1) convolutions	Restores inter-channel interaction and nonlinear representation
Scaled shortcut	Residual addition with layer scaling	Stabilizes optimization and constrains feature perturbation

**Table 2:** Heterogeneous evidence branches in RFFA.

Branch	Principal Operation	Structural Response
Position	Coordinate maps and depthwise convolution	Spatial displacement and location-sensitive anomalies
Boundary	Sobel-guided convolution	Narrow gaps, damaged contours, and edge discontinuities
Connectivity	Horizontal, vertical, and dilated depthwise convolutions	Directional interruption and abnormal conductive linkage
Texture	Large-kernel depthwise convolution and local variance	Irregular intensity fluctuations and subtle surface disturbance

**Table 3:** Structure-aware detail branches in RCF.

Branch	Principal Operation	Reconstructed Information
Boundary	Laplacian-guided depthwise convolution	Narrow gaps and contour discontinuities
Directional continuity	Horizontal, vertical, dilated convolutions	Interrupted or abnormal conductive paths
Local contrast	Local mean and deviation statistics	Weak intensity fluctuations, subtle defects

**Table 4:** Construction and partitioning of the PCB defect dataset.

Dataset Stage	Roboflow	PKU	Total
Original source images	2538	1386	3924
Retained parent images after cleaning	2488	1359	3847
Valid samples after cropping and standardization	9952	5436	15,388
Training set	7962	4357	12,319
Validation set	995	539	1534
Test set	995	540	1535

**Table 5:** Experimental environment.

Component	Configuration
Server	Dell Precision 7920 Server (Dell Inc., Round Rock, TX, USA)
System	Windows Server 2019 (Microsoft Corporation, Redmond, WA, USA)
CPU	Intel(R) Xeon(R) Gold 6133 CPU @ 2.50 GHz (Intel Corporation, Santa Clara, CA, USA)
GPU	NVIDIA GeForce RTX3090 24G (NVIDIA Corporation, Santa Clara, CA, USA)
Deep learning framework	Pytorch 2.0.0+cu118
Software Environment	Anaconda 24.5.0; Python 3.10.18; CUDA 12.4; Ultralytics 8.4.155

**Table 6:** Training parameters.

Parameter	Value
Learning rate	0.01
Image size	640 × 640
Batch size	16
Optimizer	SGD
Epoch	300
Other parameters	Ultralytics default settings
Random seed	0 for the primary experiments; 42 and 3407 for the additional repeated runs

**Table 7:** Overall ablation results and inference efficiency of the proposed CRF-YOLO.

No.	C3k2-Lite	RFFA	RCF	Shape-NWD	P/%	R/%	FPS/(f/s)	mAP @0.5/%	mAP@ 0.5:0.95/%	Params/M	GFLOPs
1					94.51	89.43	136.99	92.65	49.71	2.583	6.3
2	√				94.71	89.51	162.47	92.97	50.74	2.138	5.6
3		√			95.30	88.68	132.63	93.89	50.14	2.604	6.5
4			√ *		94.85	90.12	133.87	93.23	50.75	2.599	6.5
5				√	94.95	89.34	136.61	93.28	50.83	2.583	6.3
6	√	√			94.91	89.27	154.48	93.19	50.45	2.159	5.8
7	√	√	√		96.24	89.85	157.63	94.21	51.46	2.175	6.0
8	√	√	√	√	95.53	91.26	157.15	94.46	51.74	2.175	6.0

For configuration No. 4, RCF was evaluated without RFFA by feeding two identical copies of the raw P3 feature into its two input branches. Configurations No. 7 and No. 8 employ the standard RCF with the raw P3 feature and its RFFA-enhanced counterpart as inputs. Note: “*” denotes the controlled RCF-without-RFFA configuration, in which two identical copies of the raw P3 feature were used as the two inputs of RCF. This setting preserves the internal structure of RCF while excluding the feature enhancement produced by RFFA. Note: “√” indicates that the corresponding component is included in the model configuration, whereas a blank cell indicates that it is not included.

**Table 8:** Comparison of different lightweight feature extraction modules.

Module	mAP@0.5/%	Params/M	GFLOPs
C3k2	92.65	2.583	6.3
C3Ghost	93.00	2.304	6.0
C2fCIB	93.01	2.447	6.3
C3k2-Lite	92.97	2.138	5.6

**Table 9:** Comparison of different attention mechanisms.

Model	Attention Module	mAP@0.5/%	Params/M	GFLOPs
YOLO11n	None	92.65	2.583	6.3
YOLO11n + SE	SE	92.98	2.584	6.3
YOLO11n + CBAM	CBAM	93.33	2.584	6.3
YOLO11n + CA	CA	93.39	2.585	6.3
YOLO11n + ECA	ECA	93.25	2.583	6.3
YOLO11n + RFFA	RFFA	93.89	2.604	6.5

**Table 10:** Interaction ablation results of RFFA and RCF.

Model Configuration	mAP@0.5/%	Params/M	GFLOPs
C3k2-Lite	92.97	2.138	5.6
C3k2-Lite + RFFA	93.19	2.159	5.8
C3k2-Lite + RCF *	93.62	2.154	5.8
C3k2-Lite + RFFA + RCF	94.21	2.175	6.0

* In the controlled RCF-without-RFFA configuration, two identical copies of the raw P3 feature were used as the two inputs of RCF. This setting preserves the internal structure of RCF while excluding the feature enhancement produced by RFFA.

**Table 11:** Comparison of different bounding-box regression losses.

Loss Function	mAP@0.5/%	Params/M	GFLOPs
CIoU	92.65	2.583	6.3
MPDIoU	92.95	2.583	6.3
NWD	93.11	2.583	6.3
UIoU	92.70	2.583	6.3
Shape-NWD	93.28	2.583	6.3

**Table 12:** Comparison with mainstream object detectors on the PCB defect test set.

Model	P/%	R/%	FPS/(f/s)	mAP@0.5/%	mAP@0.5:0.95/%	Params/M	GFLOPs
YOLOv3-tiny	94.70	87.93	115.32	91.55	49.37	12.130	18.9
YOLOv5n	94.95	87.71	139.47	91.68	48.56	2.504	7.1
YOLOv6n	94.19	85.14	124.84	90.41	46.12	4.234	11.7
YOLOv8n	94.80	88.49	132.80	92.00	49.04	3.006	8.1
YOLOv9t	95.09	88.77	130.55	92.28	49.41	1.971	7.6
YOLOv10n	94.39	86.60	142.05	91.93	49.05	2.266	6.5
YOLO11n	94.51	89.43	136.99	92.65	49.71	2.583	6.3
YOLO12n	94.84	88.08	129.87	92.38	49.37	2.569	6.5
YOLO26n	95.51	88.77	148.37	93.05	50.40	2.506	5.8
CRF-YOLO	95.53	91.26	157.15	94.46	51.74	2.175	6.0

**Table 13:** Performance robustness of YOLO11n and CRF-YOLO under different random seeds.

Model	Seed	P/%	R/%	mAP@0.5/%	mAP@0.5:0.95/%
YOLO11n	0	94.51	89.43	92.65	49.71
YOLO11n	42	94.32	89.20	92.41	49.52
YOLO11n	3407	94.68	89.58	92.83	49.88
YOLO11n	Mean ± SD	94.50 ± 0.18	89.40 ± 0.19	92.63 ± 0.21	49.70 ± 0.18
CRF-YOLO	0	95.53	91.26	94.46	51.74
CRF-YOLO	42	95.38	91.08	94.29	51.58
CRF-YOLO	3407	95.72	91.45	94.67	51.91
CRF-YOLO	Mean ± SD	95.54 ± 0.17	91.26 ± 0.19	94.47 ± 0.19	51.74 ± 0.17

**Table 14:** Comparison with representative PCB-specific defect detection models.

Model	mAP@0.5 (%)	Params (M)	GFLOPs
MS-DETR	86.70	14.000	45.6
PD-YOLOv8	84.10	5.200	10.3
YOLOv8-PCB	90.60	2.500	7.1
YOLO-MobileViT	87.90	1.800	8.2
CRF-YOLO	94.46	2.175	6.0

Note: All results were obtained by retraining and evaluating the compared models on the unified PCB dataset constructed in this study. References [36,37,38,39] identify only the original sources of the corresponding model architectures.

**Table 15:** Class-wise detection performance of CRF-YOLO on the test set.

Class	Images	Instances	P/%	R/%	AP@0.5/%	AP@0.5:0.95/%
Missing hole	140	348	99.1	100.0	99.4	65.3
Mouse bite	211	547	93.9	85.7	90.7	47.5
Open circuit	232	525	93.5	87.6	92.5	44.4
Short	214	505	96.3	93.5	96.8	54.8
Spur	221	550	96.1	88.9	93.8	48.0
Spurious copper	234	530	94.4	91.9	93.4	59.4
**Overall**	**1535**	**3005**	**95.5**	**91.3**	**94.5**	**51.7**

Note: Bold values indicate the overall performance across all six defect categories.

## Data Availability

The source datasets analyzed in this study are publicly available from the Roboflow Universe platform (https://universe.roboflow.com/) and the PKU PCB defect dataset described in Reference [2] (https://doi.org/10.48550/arXiv.1901.08204).
